# TAp73 is a marker of glutamine addiction in medulloblastoma

**DOI:** 10.1101/gad.302349.117

**Published:** 2017-09-01

**Authors:** Maria Victoria Niklison-Chirou, Ida Erngren, Mikael Engskog, Jakob Haglöf, Daniel Picard, Marc Remke, Phelim Hugh Redmond McPolin, Matthew Selby, Daniel Williamson, Steven C. Clifford, David Michod, Michalis Hadjiandreou, Torbjörn Arvidsson, Curt Pettersson, Gerry Melino, Silvia Marino

**Affiliations:** 1 Blizard Institute, Barts and the London School of Medicine and Dentistry, Queen Mary University of London, London E1 2AT, United Kingdom;; 2 Department of Medicinal Chemistry, Analytical Pharmaceutical Chemistry, Uppsala University, 751 23 Uppsala, Sweden;; 3 Department of Pediatric Oncology, Hematology, and Clinical Immunology, Heinrich Heine University Dusseldorf, 40225 Dusseldorf, Germany;; 4 Department of Neuropathology, Medical Faculty, Heinrich Heine University Dusseldorf, 40225 Dusseldorf, Germany;; 5 Department of Pediatric Neuro-Oncogenomics, German Cancer Research Center (DKFZ), German Cancer Consortium (DKTK), 69120 Heidelberg, Germany;; 6 Wolfson Childhood Cancer Research Centre, Northern Institute for Cancer Research, Newcastle University, Newcastle upon Tyne NE1 7RU, United Kingdom;; 7 University College London, Institute of Child Health, London WC1N 1EH, United Kingdom;; 8 Medical Product Agency, SE-751 03 Uppsala, Sweden;; 9 Medical Research Council, Toxicology Unit, Leicester University, Leicester LE1 9HN, United Kingdom

**Keywords:** medulloblastoma, p73, glutamine, metabolomics

## Abstract

Medulloblastoma is the most common solid primary brain tumor in children. Remarkable advancements in the understanding of the genetic and epigenetic basis of these tumors have informed their recent molecular classification. However, the genotype/phenotype correlation of the subgroups remains largely uncharacterized. In particular, the metabolic phenotype is of great interest because of its druggability, which could lead to the development of novel and more tailored therapies for a subset of medulloblastoma. p73 plays a critical role in a range of cellular metabolic processes. We show overexpression of p73 in a proportion of non-WNT medulloblastoma. In these tumors, p73 sustains cell growth and proliferation via regulation of glutamine metabolism. We validated our results in a xenograft model in which we observed an increase in survival time in mice on a glutamine restriction diet. Notably, glutamine starvation has a synergistic effect with cisplatin, a component of the current medulloblastoma chemotherapy. These findings raise the possibility that glutamine depletion can be used as an adjuvant treatment for p73-expressing medulloblastoma.

## Abstract (precis)

Niklison-Chirou et al. identify a proportion of non-WNT medulloblastoma in which p73 sustains cell growth and proliferation via regulation of glutamine metabolism.

Medulloblastoma (MB) is the most common primary pediatric brain tumor and is a major cause of mortality and morbidity in pediatric oncology (Louis et al. 2007). Current treatment strategies include a combination of surgery, radiotherapy, and chemotherapy, which have achieved 70%–80% 5-yr survival rates; however, recurrence is common and often proves fatal (Hill et al. 2015). In addition, both the brain exposure to ionizing radiation and the systemic exposure to chemotherapy agents can induce severe side effects, leading to mental and physical disability of the affected children.

MB is a heterogeneous tumor with molecularly defined subgroups that arise from different cerebellar progenitor cells, albeit often showing rather similar histological features (Corno et al. 2012). MBs have been classified recently into four molecular groups; namely, the WNT group, the Sonic Hedgehog (SHH) group, group 3 (G3), and group 4 (G4) (Taylor et al. 2012). The discovery that mutant TP53 plays an important role in SHH MB pathogenesis led to the further dissection of the SHH group into TP53 wild-type and TP53 mutant subgroups, the latter being associated with a poor outcome (Ramaswamy et al. 2015). Emerging evidence suggests that each group may require specific therapeutic strategies (Northcott et al. 2012), and recent studies demonstrated that WNT MB have anomalous vascularization and show increased hemorrhaging (Phoenix et al. 2016).

The p53 family comprises three members (p53, p63, and p73) (Boominathan 2010), and its role in promoting cell death and senescence has been described extensively. Importantly, p63 plays a particular role in the development of the epidermis (Vanbokhoven et al. 2011), and p73 is essential for the development of the central nervous system (CNS) (Yang et al. 2000). Indeed, p73 is highly expressed in the hippocampus, cortex, and cerebellum during embryonic stages of CNS development; its expression then decreases after birth and is restricted to the neural stem cell (NSC) niches in the adult brain (Pozniak et al. 2002). p73-deficient mice show prominent brain malformations and reduced NSC proliferation (Talos et al. 2010). Unlike p53, p73 is not lost but rather is frequently overexpressed in cancer. Accordingly, p73 is overexpressed in MB tumors and cell lines even though its role in these tumors is still unclear (Zitterbart et al. 2007).

At the molecular level, *p73* is transcribed from two different promoters into proteins that either retain (TAp73) or lack (ΔNp73) the transactivation domain. TAp73 is able to activate p53-responsive genes and induce apoptosis (Zhu et al. 1998), although TAp73 also has distinct transcriptional targets (Allocati et al. 2012). In contrast, ΔNp73 displays an anti-apoptotic effect (Dulloo et al. 2010). Recent studies have shown that p73 plays an important role in the regulation of metabolic pathways. TAp73 enhances the pentose phosphate pathway flux (Jiang et al. 2013), activates serine biosynthesis (Amelio et al. 2014b), and controls glutaminolysis (Velletri et al. 2013). TAp73 regulates the mitochondrial respiration by inducting cytochrome *c* oxidase (Rufini et al. 2012), and its depletion results in decreased oxygen consumption and ATP levels with increased reactive oxygen species (ROS) levels. p73 is also a major transcriptional regulator of autophagy (He et al. 2013) and is activated when mTOR is inhibited (Rosenbluth and Pietenpol 2009). Consistent with these data, TAp73 knockout mice show premature aging and senescence (Rufini et al. 2012).

Metabolic adaptation has emerged recently as a hallmark of cancer and a promising therapeutic target (Hanahan and Weinberg 2011). Accordingly, highly proliferating cancer cells must adapt their metabolism in order to produce enough energy and mass to replicate. The first step of adaptation is through enhanced aerobic glycolysis, which allows cells to metabolize glucose to lactate instead of pyruvate (Warburg 1956). Aerobic glycolysis in cancer cells is essential for tumor progression and, in MB, has been estimated to account for 60% of ATP production (Moreno-Sanchez et al. 2009).

In addition to the dependency on aerobic glycolysis, cancer cells exhibit other metabolic characteristics such as increased fatty acid synthesis and addiction to glutamine. Some cancer cells show glutamine addiction regardless of the fact that glutamine is a nonessential amino acid and one that can be synthesized from glucose (DeBerardinis and Cheng 2010). Glutamine is used by the cancer cells to synthetize amino acid precursors and in maintaining activation of TOR kinase (Ahluwalia et al. 1990). Moreover, glutamine is the primary mitochondrial substrate and is required to maintain mitochondrial membrane potential and support the NADPH production needed for redox control and macromolecular synthesis (Wise and Thompson 2010). Importantly, MB metabolism exhibits a high dependency on aerobic glycolysis and lipogenesis through the activation of hexokinase 2 and fatty acid synthase (Gershon et al. 2013; Tech et al. 2015). Additionally, MBs limit protein translation through activation of eukaryotic elongation factor 2 kinase to restrict energy expenditure (Leprivier et al. 2013). This difference between cancer and normal cells suggests that targeting metabolic dependence could be a selective approach to treat cancer patients.

In this study, we set out to investigate the metabolic pathways regulated by p73 in MB by means of genome-wide transcriptome and metabolome analysis in MB cell lines and patient-derived MB cells with subsequent biochemical and functional validation in vitro and in vivo in a xenograft mouse model.

## Results

### TAp73 is overexpressed in MB and controls proliferation in MB cell lines and patient-derived primary cells

**Figure 1.**
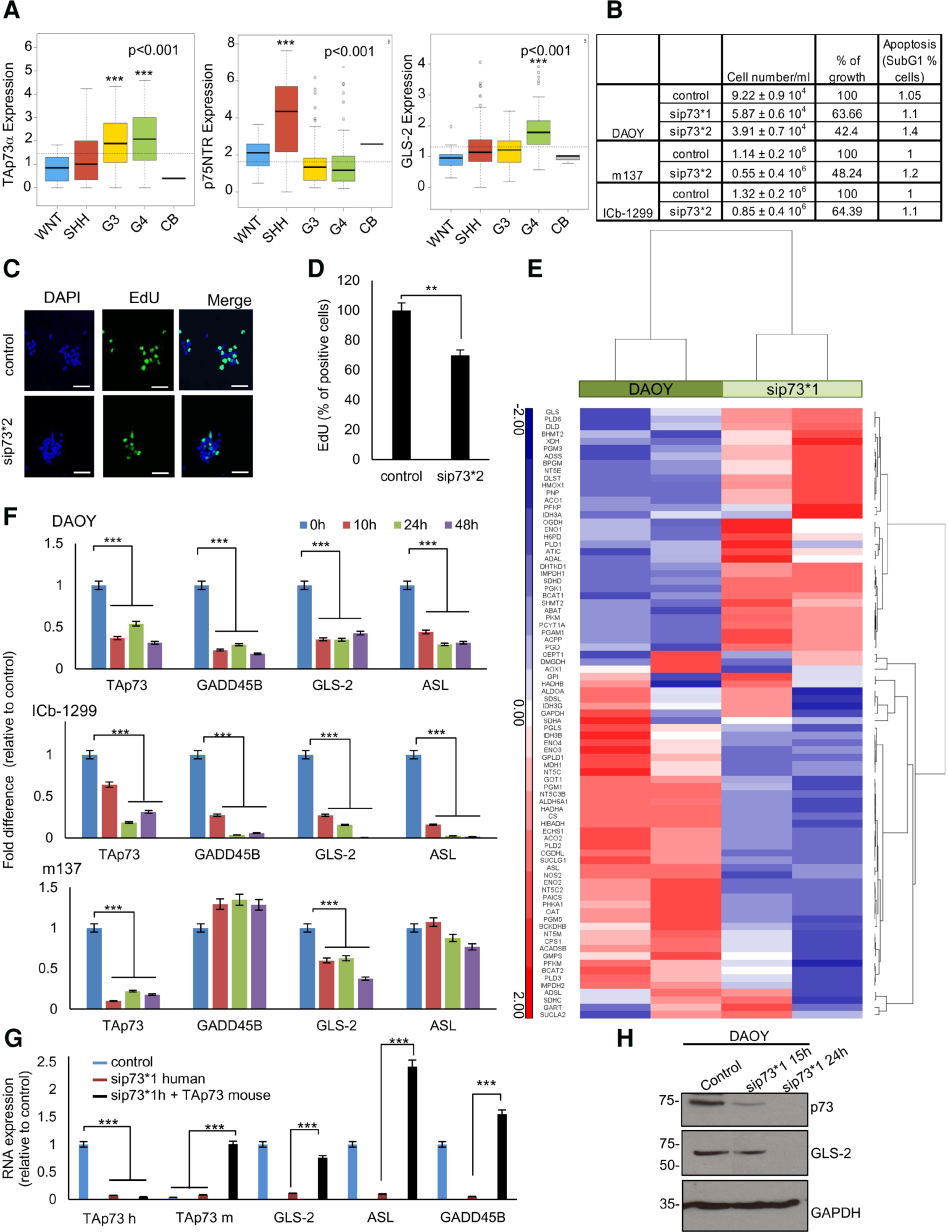
p73 is overexpressed in MB and regulates GLS-2 expression. (*A*) Box plot representation of *TAp73α*, *p75NTR*, and *GLS-2* expression levels (FPKM [fragments per kilobase of transcript per million mapped reads]) across a cohort of 240 primary MBs. Patient numbers per group were 28 WNT, 58 SHH, 59 G3, 95 G4, and three CB. (***) *P* < 0.001. (*B*) DAOY, m137, and ICb-1299 cells were transiently transfected with scramble or sip73 (sip73*1 ID 2671; sip73*2 ID 115666). After 48 h, cells under different treatments were counted in triplicate. For apoptosis assessment (percentage of cells in SubG1), cells were stained with propidium iodide (PI) and analyzed by FACS. (*C*) Confocal microscope images of ICb-1299 scramble cells or sip73*2 after 48 h. We assessed cell proliferation by EdU incorporation. Bar, 20 µm. (*D*) Quantification of EdU staining in ICb-1299 scramble cells and sip73*2. Columns indicate mean ± SEM. *n* = 3. (**) *P* < 0.001. (*E*) Heat map representation of the most significantly up-regulated or down-regulated genes after sip73*1. Genes involved in metabolism were plotted. Genes were identified using Molecular Signatures Database (MSigDB) and plotted to highlight differences in expression of three *z*-scores or greater between the two groups. (Red) Relative up-regulation; (blue) relative down-regulation. (*F*) MB cells (DAOY, ICb-1299, and m137) were transiently transfected with scramble or sip73*1 for 10, 24, and 48 h. Columns indicate mean ± SEM. *n* = 3. (***) *P* < 0.0001. (*G*) DAOY cells were transiently transfected with scramble or sip73*1 human or sip73*1 human plus TAp73 mouse for 10 h. RT–PCR was performed for *TAp73* human, *TAp73* mouse, *GLS-2*, *ASL*, and *GADD45B* genes. Columns indicate mean ± SEM. *n* = 3. (***) *P* < 0.0001. (*H*) DAOY cells were transfected with scramble or sip73*1. Western blot showing p73 and GLS-2 protein levels after silencing. GAPDH was used as a loading control.

p73 was reported to be overexpressed in MB (Zitterbart et al. 2007), although it was unclear which p73 isoforms were expressed. To clarify this, we analyzed RNA sequence data derived from 240 clinically characterized human MBs. Significant overexpression of TAp73α was found in G4 and G3 MBs as compared with normal cerebella, with high expression levels found in SHH MBs and very low levels found in WNT MBs (Fig. 1A). TAp73β, ΔNp73α, and ΔNp73β isoforms were not significantly expressed in MB (Supplemental Fig. S1A). Next, we looked at the expression of *p75NTR* and *GLS-2*, two well-characterized TAp73 target genes important for brain development and glutamine metabolism, respectively (Niklison-Chirou et al. 2013; Velletri et al. 2013). Consistent with the TAp73 levels, significant up-regulation of *GLS-2* was found in the G4 MBs, while the highest expression of *p75NTR* was detected in SHH MBs (Fig. 1A). Overall, these analyses demonstrate that the most aggressive subgroups of MB express high levels of *TAp73* mRNA.

Next, we analyzed the expression of *p73* in a range of human patient-derived primary MB cells and cell lines. We found the highest level in lCb-1299 (primary human G4 MB) and intermediate levels in primary SHH MB cells (m137 and m692) and the DAOY cell line, with no expression detected in the UW228-2 cell line (Supplemental Fig. S1B). We show at the RNA and protein levels that the only isoform expressed in MB cells is TAp73, as a single band of 75 kDa was detected in the Western blot (Supplemental Fig. S1B,C). RT–PCR analysis of *TAp73*, *p75NTR*, and *GLS-2* in lCb-1299, m137, m692, DAOY, and UW228-2 cells revealed up-regulation of all genes in MB cells expressing p73 (Supplemental Fig. S1D–F), in keeping with the results of the genome-wide analysis on human primary tumors (Fig. 1A).

To explore the role of TAp73 in MB, we transiently knocked down p73 (p73KD) in DAOY, m137, and ICb-1299 cells. Western blot and RT–PCR confirmed p73KD in all cells (Supplemental Fig. S1G,H) and showed that the levels of the other p53 family members (p53 or p63) were not affected (Supplemental Fig. S1G). Silencing p73 strongly reduced cell growth in DAOY, m137, and ICb-1299 (Fig. 1B). Importantly, we observed a 30% reduction in cell proliferation in ICb-1299 cells, where only a partial (44% of p73 levels) knockdown could be obtained (Fig. 1C,D).

These results show that p73 is essential for MB cell growth and proliferation.

### TAp73 regulates GLS-2 in MB cells

p73 regulates the expression of many target genes involved in cell metabolism, DNA repair, or apoptosis. To dissect the molecular pathways mediating p73's role in MB, we performed a differential gene expression analysis in DAOY comparing p73KD cells with control cells at 48 h after silencing. RNA sequencing (RNA-seq) analysis revealed that a large number of genes involved in metabolism and/or stress pathways were differentially expressed upon p73KD, and unsupervised hierarchical clustering (HCL) demonstrated that DAOY p73KD cluster apart from control cells, suggesting that a distinct transcriptome-wide gene signature was associated with the silencing of p73 (Fig. 1E, genes involved in metabolism; Supplemental Fig. S1I, genes involved in stress pathways). Because we were interested in identifying genes that are specifically transactivated by p73, we focused our attention on genes that are down-regulated in the absence of p73.

We selected 14 genes (>1.5 fold change; *P* < 0.05) for validation as p73 target genes: *GADD45B*, *GLS-2*, *ASL*, *SFN*, *PFKM*, *ACO2*, *NOS2*, *PHKA1*, *NBN*, *ENO*, *AKT2*, *SDHA*, *CS*, and *RAD50* (Fig. 1E; Supplemental Fig. S1I,J). p73KD was repeated in DAOY cells, and expression of the target genes was analyzed at three different time points: 10, 24, and 48 h after silencing. We detected an early down-regulation (10 h after knockdown) of *GADD45B*, *GLS-2*, and *ASL* (Fig. 1F; Supplemental Fig. S1K). To assess whether this molecular convergence was retained in patient-derived primary MB cells expressing p73, the expression of *GADD45B*, *GLS-2*, and *ASL* was assessed in lCb-1299 and m137 after p73KD. In agreement with our previous results, we observed an early down-regulation of *GLS-2* in both cell lines upon p73KD (Fig. 1F). *GADD45B* and *ASL* were down-regulated only in lCb-1299. Importantly, re-expression of a mouse TAp73 rescued the effect of p73KD on the expression levels of *GADD45B*, *GLS-2*, and *ASL*, confirming that they were primary p73 targets in DAOY cells (Fig. 1G). Down-regulation of GLS-2 after p73KD was confirmed at the protein level in DAOY cells (Fig. 1H).

These results show that GLS-2 is a conserved TAp73 target gene in MB cells derived from non-WNT MB subgroups.

### TAp73 is a critical cellular component for mitochondrial bioenergetics in MB cells

**Figure 2.**
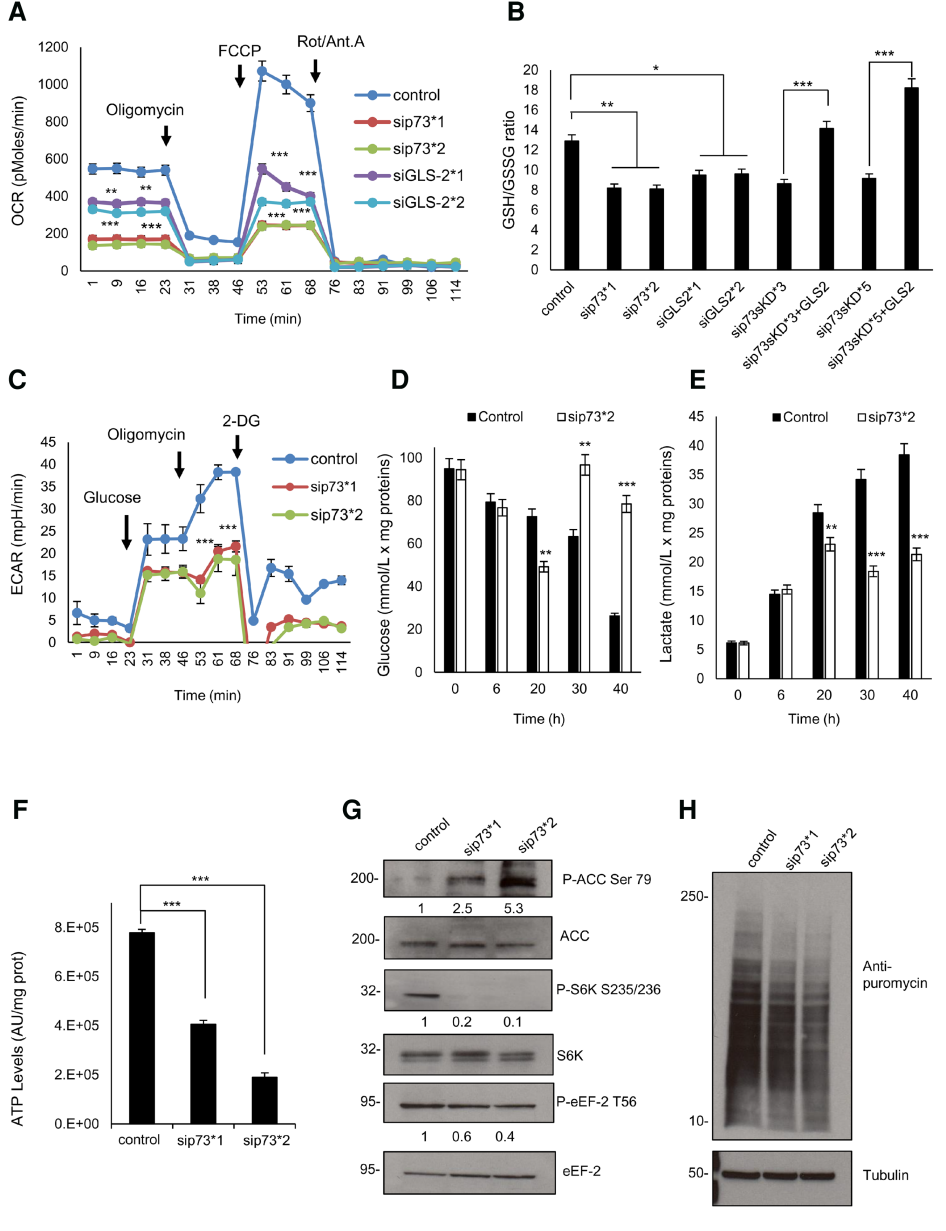
p73KD induces mitochondrial defects and activates AMPK signaling. (*A*) DAOY cells were transfected with scramble, sip73 (sip73*1, 2671; sip73*2, 115666), or siGLS-2 (siGLS-2*1, s25941; siGLS-2*2, s223735). OCR in response to a mitochondrial stress test was recorded to construct functional bioenergetic profiles. A minimum of five different samples was analyzed for each group. (**) *P* < 0.001; (***) *P* < 0.0001, unpaired two-sided *t*-test. Data from one representative experiment out of three are shown. (*B*) The reduced and oxidized glutathione ratio was measured in DAOY cells after transient transfection with scramble, sip73*1, sip73*2, siGLS-2*1, and siGLS-2*2 for 24 h. DAOY cells were infected with an empty vector or with shRNA (p73sKD*3 and p73sKD*5) alone or transfected with Flag-GLS-2-expressing vector for 48 h. *n* = 3. Data were mean ± SD. (*) *P* < 0.01; (**) *P* < 0.001; (***) *P* < 0.0001. (*C*) DAOY cells were transfected with scramble or sip73 (sip73*1, 2671; sip73*2, 115666). Extracellular acidification rate (ECAR) was measured in response to a mitochondrial stress test. A minimum of five different samples was analyzed for each group. (***) *P* < 0.0001, unpaired two-sided *t*-test. Data from one representative experiment out of three are shown. (*D*,*E*) DAOY cells were transfected with scramble or sip73*2. After 8 h of transfection, the medium was changed, and samples were collected after 6, 20, 30, and 40 h. The results show glucose consumption (*D*) or lactate production (*E*). Columns indicate mean ± SEM. *n* = 4. (**) *P* < 0.001; (***) *P* < 0.0001. (*F*) DAOY cells were transiently transfected with scramble, sip73*1, or sip73*2. After 48 h, cells were collected, and ATP levels were determined. Columns indicate mean ± SEM. *n* = 4. (***) *P* < 0.0001. (*G*) DAOY cells were transfected with scramble, sip73*1, or sip73*2. After 48 h a Western blot was performed for ACC, P-ACC, S6K, P-S6K, eEF-2, and P-eEF-2. Intensity analysis is shown *below* each lane; scramble was considered 1. (*H*) DAOY cells were transfected with scramble, sip73*1, or sip73*2. After 48 h, cells were treated for 1 h with 2.5 mg/mL puromycin. Next, cells were collected, and a Western blot of lysed cells was probed with a primary anti-puromycin antibody. Tubulin was used as a loading control. (2-DG) 2-deoxy-D-glucose.

The importance of glutamine as a nutrient in cancer relies on its suitability as a substrate for the mitochondrial TCA cycle (Hensley et al. 2013). Since we observed a strong reduction of GLS-2 after p73KD, we measured the mitochondria oxygen consumption rate (OCR) as an indicator of the mitochondrial function. A functional bioenergetics profile of DAOY p73KD as compared with control cells is shown in Figure 2A. We measured the OCR in response to sequential treatment with oligomycin, carbonyl cyanide-4-(trifluoromethoxy)phenylhydrazone (FCCP), and rotenone/antimycin A. We observed a strong decrease in the basal respiration, ATP production, and spare respiratory capacity upon p73KD, which suggested profound respiratory defects after p73KD (Fig. 2A). Similar results were obtained after GLS-2KD (Fig. 2A; Supplemental Fig. S2A). These findings were validated in patient-derived primary MB cells (m137 and ICb-1299), where similar results were obtained (Supplemental Fig. S2B,C).

Next, we performed a stable p73KD (p73sKD) with two different shRNAs: p73sKD*3 and p73sKD*5. The reduction of p73 protein levels in steady state was confirmed by immunoblotting (Supplemental Fig. S2D). Importantly, re-expression of GLS-2 in a p73sKD setting (p73sKD*5) rescued the OCR levels (Supplemental Fig. S2E–G).

While assessing whether p73KD induces mitochondrial dysfunction, we observed a strong mitochondrial hyperpolarization in DAOY cells upon p73KD (Supplemental Fig. S2H). Because mitochondrial hyperpolarization has been related to ROS production (Giovannini et al. 2002) and because glutamine is a precursor of glutathione synthesis (GSH; the major antioxidant within the cells), we measured the redox status of DAOY cells upon p73KD or in our stable p73sKD*3 or p73sKD*5 or after GLS-2KD as assessed by the GSH/GSSG ratio (Fig. 2B). A decreased ratio of reduced glutathione to glutathione disulfide was observed after p73KD or p73sKD or after siGLS-2, confirming that after p73KD or GLS-2KD, DAOY cells are under oxidative stress. Importantly, this effect was completely rescued after GLS-2 overexpression (Fig. 2B), indicating that GLS-2 is an important p73 target gene for balancing the redox status of the cells.

Since 60% of ATP production in MB cells is generated by aerobic glycolysis (Moreno-Sanchez et al. 2009), we measured the extracellular acidification rate (ECAR) in DAOY cells upon p73KD (Fig. 2C). The results show a robust decline (50%) in lactate production after p73KD compared with control cells in response to oligomycin treatment, suggesting a reduced ability to increase glycolytic flux under conditions of increased ATP demand.

Next, we set out to confirm these results by measuring the kinetics of glucose uptake and lactate production in DAOY upon p73KD at different time points. In control cells, the levels of glucose decreased steadily over time, with an increased lactate production observed in parallel, whereas an inhibition of glucose uptake and production of lactate was observed after 20 h in DAOY p73KD (Fig. 2D,E). Importantly, glucose levels increased in DAOY p73KD, indicating that the lack of TAp73 induced gluconeogenesis in the longer term. Gluconeogenesis is a common process in tumor cells in which cells can derive energy from ketone bodies, which are converted to acetyl-CoA and shunted into the TCA cycle.

Because oxidative phosphorylation and aerobic glycolysis are the two main sources of cellular energy, we measured ATP levels in DAOY, m137, and lCb-1299 cells. In line with our previous results, a strong reduction in the ATP levels was observed upon p73KD in DAOY, m137, and ICb-1299 cells (Fig. 2F; Supplemental Fig. S2I). Similar results were observed upon GLS-2KD (Supplemental Fig. S2I).

Taken together, these data suggest that p73 is essential for the major energy-producing pathways and for maintenance of the redox balance of the cells through the generation of glutathione via the regulation of GLS-2.

### p73 sustains activation of the mTOR pathway by inhibiting AMPK activation in MB

AMPK plays a role as a master regulator of cellular energy, is activated when cellular ATP levels drop, and initiates a cellular reprogram that allows the cell to adapt to the energetic stress (Hardie et al. 2012). We observed a robust activation of AMPK, as assessed by phosphorylation of its direct target, acetyl-CoA carboxylase (ACC), in DAOY after p73KD (Fig. 2G). An important mediator of the AMPK response is the mTOR pathway, which is inhibited by AMPK (Liu et al. 2012). We show here inhibition of the mTOR pathway, as shown by reduced phosphorylation of its downstream targets, the protein S6 kinase (S6K) and eukaryotic translation elongation factor 2 (eEF-2), upon p73KD (Fig. 2G). mTOR is a nutrient sensor, by which glutamine and leucine levels stimulate protein synthesis via signaling through the mTOR complex. Conversely, mTOR inhibition leads to reduction in the rate of the global protein production (Zhao et al. 2015). In order to determine whether p73 supports protein synthesis in DAOY cells, we silenced p73 in DAOY cells and then treated the cells for 1 h with puromycin. The incorporation of puromycin in the newly synthesized proteins was assessed with an anti-puromycin antibody. We found reduced protein synthesis of ∼50% in DAOY p73KD (Fig. 2H; Supplemental Fig. S2J). These data suggest that p73 modulates mTOR activation through reduction in AMPK activity in MB cells.

### Profound metabolic changes in MB cells after p73KD

**Figure 3.**
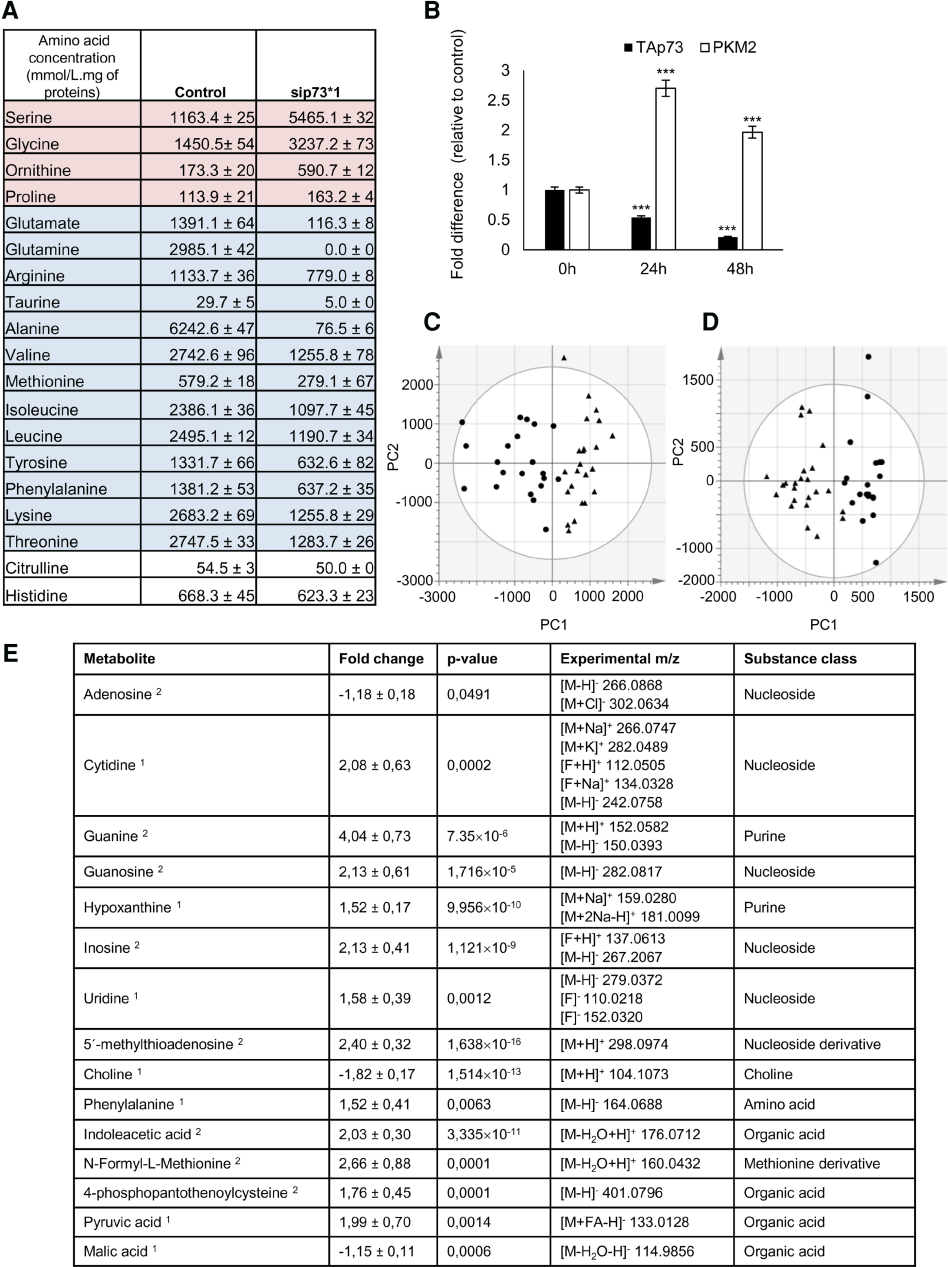
p73 regulates metabolic pathways in MB. DAOY cells were transfected with scramble or sip73*1 (ID 2671). (*A*) After 48 h, the medium was collected, and amino acid levels were quantified. *n* = 3. (*B*) After 0, 24, and 48 h, cells were collected, and RT–PCR was performed for *TAp73α* and *PKM2* genes. Columns indicate mean ± SEM. *n* = 3. (***) *P* < 0.0001. (*C*,*D*) Liquid chromatography hyphenated to high-resolution mass spectrometry (LC-HRMS) metabolic profiling of DAOY scramble (●) and sip73*1 cells (▴). PCA score plots of the first two components: PC1 and PC2. The data were normalized to median fold change and pareto scale. (*C*) Positive ionization mode (R2X = 0.602 and Q2 = 0.137). (*D*) Negative ionization mode (R2X = 0.761 and Q2 = 0.355). (*E*) List of the affected metabolites after p73KD in DAOY cells. (Note 1) Confidently identified metabolites; identification was based on retention time, exact mass, and fragmentation as compared with an analytical standard that was analyzed under the same conditions. (Note 2) Putatively annotated metabolites; annotation was based on exact mass and fragmentation as compared with available reference spectra.

Cellular metabolism is characterized by many parameters, including nutrient uptake or metabolite secretion rates (Buescher et al. 2015). We measured amino acid levels in the medium of DAOY p73KD as compared with controls 48 h after silencing. We show that the lack of p73 reduces the levels of 14 amino acids and increases the levels of four. Alanine, glutamine, and glutamate are the most noticeably reduced amino acids (Fig. 3A). Interestingly, the main source of carbon skeletons used for gluconeogenesis are lactate and the amino acids alanine and glutamine; thus, these data are in agreement with our previous observation that p73KD induces gluconeogenesis (Fig. 2D).

The four amino acids augmented in the medium are serine, glycine, ornithine, and proline (Fig. 3A). Serine and glycine are biosynthetically linked and are essential for one-carbon metabolism (Amelio et al. 2014a). Since these amino acids show increased levels in the absence of the p73, it is likely that this represents a compensatory effect not directly linked to p73. We confirmed these results by measuring the kinetics of serine and glycine production (Supplemental Fig. S3A,B); we observed a significant increase in serine/glycine synthesis after 20 h of p73KD. Importantly, serine can be used by cancer cells for the de novo synthesis of ATP (Maddocks et al. 2016).

Next, we measured the expression of pyruvate kinase isozyme M2 (PKM2), an enzyme characterized by a low affinity to its substrate, phosphoenolpyruvate (PEP), which is almost inactive at physiological PEP concentrations. High RNA level of PKM2 was observed in DAOY cells after 24 and 48 h of p73KD (Fig. 3B). Importantly, the presence of PKM2 induces accumulation of glycolytic intermediates above pyruvate kinase and therefore increases the synthesis of serine/glycine or the pentose phosphate pathway flux.

Following this, we used liquid chromatography hyphenated to high-resolution mass spectrometry (LC-HRMS)-based untargeted metabolite profiling to study potential differences in the intracellular polar metabolome (endogenous molecules with a molecular weight >1000 Da) in DAOY p73KD as compared with controls 48 h after silencing. A clear difference between the two conditions was found (Fig. 3C,D), indicating that silencing of p73 induces a strong difference in the polar metabolites of the cells. The absence of p73 in DAOY cells induces a significant reduction of adenosine, malic acid, and choline as well as several phospholipids. The reduction in adenosine observed is in agreement with the reduction in ATP levels observed after p73KD (Fig. 2F; Supplemental Fig. S2I). Importantly, we observed a strong reduction in choline and phospholipids (Fig. 3E), both of which can function as an energy source since they can be degraded to produce acetyl-CoA, and the reduction in malic acid is in line with our observation that the activity of the TCA cycle was reduced after p73KD.

Importantly, the levels of cytidine, guanine, guanosine, hypoxanthine, inosine, uridine, phenylalanine, indoleacetic acid, N-formyl-L-methionine, 4-phosphopantohenoylcysteine, and pyruvic acid were significantly (*P* < 0.05) up-regulated (Fig. 3E). We performed a pathway analysis using the Web-based software MetaboAnalyst (http://www.metaboanalyst.ca). We found that several metabolites were related and observed an up-regulation of purine metabolism. These data are in line with our observation that after p73KD, there is an increase in serine/glycine synthesis, whereby serine can contribute to the purine metabolism by providing one-carbon units (Maddocks et al. 2016).

During gluconeogenesis, lactate and alanine are consumed to produce pyruvate—hence the strong reduction in alanine and the increased levels of pyruvate that we observed in DAOY p73KD. Pyruvate is oxidized to feed into the gluconeogenic pathway (Supplemental Fig. S3C).

In summary, silencing of p73 in DAOY cells induces a profound metabolic alteration in which the cells try to compensate for the lack of energy due to reduced glutamine metabolism through a compensatory mechanism. Instead, lactate and pyruvate intermediates are used to feed into the gluconeogenic pathway. This process allows the cells to produce large amounts of serine/glycine and synthetize intermediates of the purine pathways and nucleosides, probably to synthetize ATP de novo.

### TAp73 expression is a biomarker of glutamine addiction in MB cells

Glutamine is an abundant and versatile nutrient that participates in energy formation, redox homeostasis, and macromolecular synthesis in cancer cells (Altman et al. 2016). These characteristics make glutamine metabolism an appealing target for new clinical strategies. Importantly, some cancers display addiction to glutamine despite the fact that glutamine is a nonessential amino acid (Wise and Thompson 2010).

**Figure 4.**
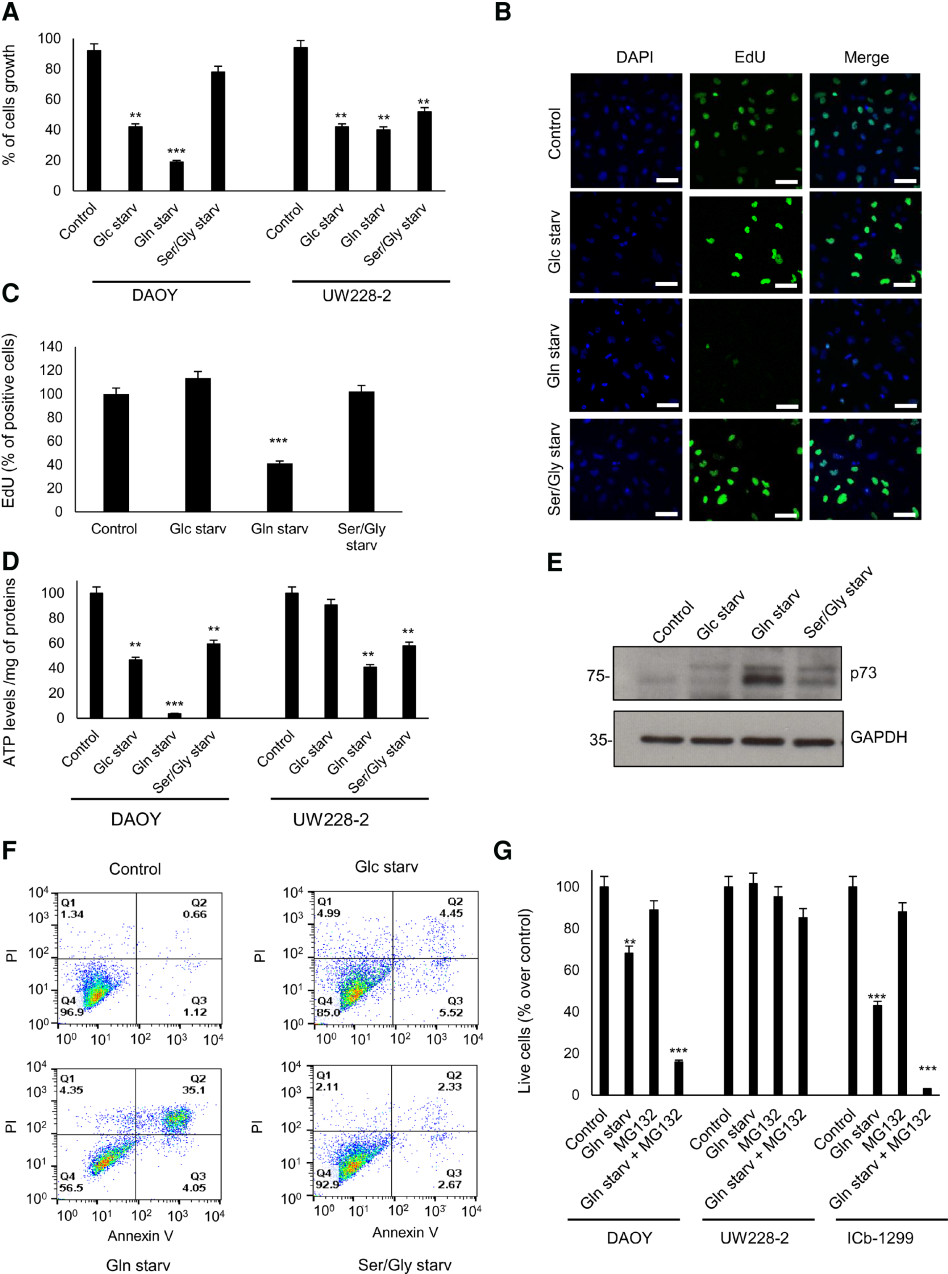
p73 is a marker of glutamine addiction in MB. (*A*) DAOY and UW228-2 cells were cultured for 24 h under normal medium, glucose starvation (Glc starv), glutamine starvation (Gln starv), and serine/glycine starvation (Ser/Gly starv) conditions. DAOY and UW228-2 cells were collected, and cell numbers were determined. Data are represented as mean ± SD. *n* = 3. (**) *P* < 0.001; (***) *P* < 0.0001. (*B*) Representative images of DAOY cells under normal medium, glucose starvation, glutamine starvation, and serine/glycine starvation for 15 h. Cells were stained with the proliferation marker EdU (green) and nuclear marker DAPI (blue). (*C*) Histogram shows mean fluorescence intensities of EdU in DAOY cells under normal medium, glucose starvation, glutamine starvation, and serine/glycine starvation for 15 h. *n* = 300 cells. Data are represented as mean ± SD. (***) *P* < 0.0001. (*D*) DAOY and UW228-2 cells were cultured for 24 h under normal medium, glucose starvation, glutamine starvation, and serine/glycine starvation conditions. DAOY and UW228-2 cells were collected, and ATP levels were determined. Data were mean ± SD. *n* = 3. (**) *P* < 0.001; (***) *P* < 0.0001. (*E*) DAOY cells were cultured for 24 h under normal medium, glucose starvation, glutamine starvation, and serine/glycine starvation. Next, cells were collected, and a Western blot of lysed cells was performed against p73. GAPDH was used as a loading control. (*F*) DAOY cells were cultured for 20 h under normal medium, glucose starvation, glutamine starvation, and serine/glycine starvation. Apoptosis was measured with Annexin V-FITC and PI for flow cytometry analysis. Images are representative of at least three independent experiments. (*G*) DAOY, UW228-2, and ICb-1299 cells were cultured for 20 h under normal medium, glutamine starvation, and glutamine starvation plus 10 µM MG132. Apoptosis was measured with Annexin V-FITC and PI for flow cytometry analysis. *n* = 3. Data are represented as mean ± SD. (**) *P* < 0.001; (***) *P* < 0.0001.

Nutrient deprivation is a strong stress with dire consequences on cell viability and energy status (Leprivier et al. 2013). Therefore, we investigated whether removal of the amino acid glutamine or serine/glycine or glucose induced a differential response between MB cells expressing p73 and those without p73. First, we measured the impact on cell growth after glucose, glutamine, and serine/glycine starvation. Cell numbers were recorded in DAOY and UW228-2 cultures after 24 h under starvation as examples of a p73-expressing and non-p73-expressing line, respectively. In DAOY cells, we observed proliferation arrest under glucose starvation, a significant reduction of the cell number under glutamine starvation, and no significant effect under serine/glycine starvation (Fig. 4A). Likewise, the three starvation conditions were able to stop cell proliferation of UW228-2 cells (Fig. 4A). Because we observed a reduction in cell number in DAOY cells after glutamine starvation, we hypothesize that this could be due to cell death. To validate these results, we measured cell proliferation of DAOY and UW228-2 cells under the three starvation conditions. We found that only glutamine starvation induces a robust inhibition of cell proliferation in DAOY cells after 15 h of treatment, as assessed by EdU incorporation (Fig. 4B,C).

It was reported that nutrient deprivation induces autophagy (Filomeni et al. 2015); therefore, we measured the levels of LC3-II by FACS analysis in DAOY and UW228-2 cells in control medium and after 24 h under glucose, glutamine, or serine/glycine starvation. No increase in LC3-II levels was observed (Supplemental Fig. S4A).

Next, we assessed whether glucose, glutamine, or serine/glycine was essential for ATP production in DAOY and UW228-2 cell lines. Cells were subjected to starvation for 24 h followed by measurement of the ATP levels (Fig. 4C). We show a 96% reduction in the ATP levels in DAOY cells after glutamine starvation, while no changes were seen in UW228-2, suggesting that the cell line expressing p73 had a glutamine addiction phenotype (Fig. 4D).p73 protein levels are usually maintained low by rapid proteasome degradation; however, DNA damage and different stress signals may trigger the stabilization of p73. Therefore, we measured p73 levels in DAOY cells after 18 h under the three starvation conditions (Fig. 4E). We observed up-regulation of p73 only under glutamine starvation. Importantly, glutamine starvation of DAOY cells for 4, 6, and 8 h concomitantly with MG132, a proteasome inhibitor that reduces the degradation of ubiquitin-conjugated proteins, induced enhancement of the p73 stabilization (Supplemental Fig. S4B). Since increased levels of p73 can induce apoptosis (Asher et al. 2005), we measured apoptosis with Annexin/PI in DAOY and UW228-2 cells under the different starvation conditions. After 36 h of starvation, only DAOY cells under glutamine starvation show a strong apoptosis response (Fig. 4F), while UW228-2 did not show apoptosis under the three starvation conditions (Supplemental Fig. S4C).

Next, we challenged DAOY, UW228-2, and the primary G4 cells (lCb-1299) with glutamine starvation or glutamine starvation plus MG132. We show a strong synergistic effect in inducing cell death in DAOY and lCb-1299 after glutamine starvation plus MG132, while no effect was observed in UW228-2 cells (Fig. 4G).

Subsequently, we analyzed the kinetics of glucose uptake and lactate production in DAOY cells in control medium and under glutamine starvation. We observed that glucose levels decreased steadily over time in DAOY cells in control medium, with an increased lactate production observed in parallel; an inhibition of glucose uptake and production of lactate was observed after 20 h in DAOY under glutamine starvation (Supplemental Fig. S4D,E). Importantly, we were unable to detect the induction of gluconeogenesis under glutamine starvation conditions. Therefore, we measured serine and glycine levels in the medium of DAOY cells in control medium or under glutamine starvation conditions (Supplemental Fig. S4F,G). We observed an initial drop in serine and glycine levels, but, at a later time point (40 h), the levels were equal to control. These data suggest that under glutamine starvation conditions, DAOY cells are not able to induce a metabolic reprogramming to allow them to adapt and survive under glutamine starvation.

These data strongly support our hypothesis that expression of p73 predicts a phenotype of glutamine addiction in MB cells.

### Synergistic effect of glutamine starvation and cisplatin in MB cells expressing p73

**Figure 5.**
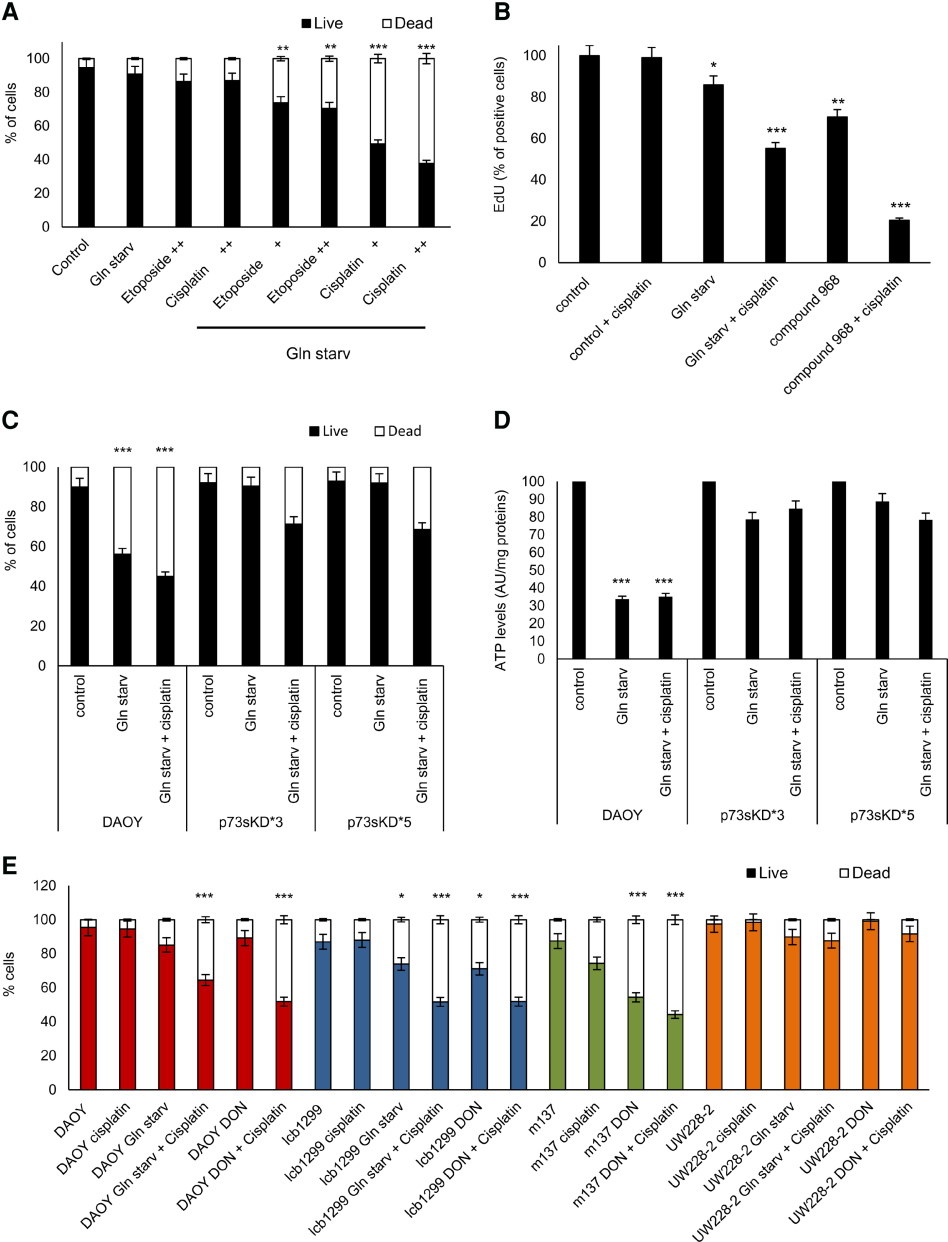
Cotreatment of cisplatin plus glutamine starvation induces a synergic apoptotic effect in MB. (*A*) DAOY cells were cultured for 18 h under normal medium or glutamine starvation with or without etoposide ([+] 6.8 nM; [++] 13.6 nM) or cisplatin ([+] 16.6 nM; [++] 33.3 nM). Apoptosis was measured with Annexin V-FITC and PI for flow cytometry analysis. Data are represented as mean ± SD. *n* = 3. (**) *P* < 0.001; (***) *P* < 0.0001. (*B*) Histogram shows mean fluorescence intensities of EdU in ICb-1299 cells under control medium, glutamine starvation, or 4 µM compound 968 with or without 16.6 nM cisplatin for 18 h. (*) *P* < 0.01; (**) *P* < 0.001; (***) *P* < 0.0001. (*C*,*D*) DAOY cells were infected with an empty vector or shRNA p73sKD*3 and p73sKD*5. Cells were incubated for 36 h under normal medium, glutamine starvation, or glutamine starvation plus 16.6 nM cisplatin. Apoptosis was measured with Annexin V-FITC and PI by flow cytometry (*C*) or ATP levels (*D*). Data are represented as mean ± SD. *n* = 3. (***) *P* < 0.0001. (*E*) Apoptosis was determined in DAOY, ICb-1299, m137, and UW228-2 cells. Cells were treated with 16.6 nM cisplatin, glutamine starvation, glutamine starvation plus 16.6 nM cisplatin, 0.9 mM 6-diazo-5-oxo-L-norleucine (DON), or DON plus 16.6 nM cisplatin. The cells were collected and stained with Annexin V-FITC and PI for flow cytometry analysis. Data are represented as mean ± SD. *n* = 3. (*) *P* < 0.01; (***) *P* < 0.0001.

Cisplatin and etoposide are effective in MB patients (Evans et al. 2009); therefore, we treated DAOY cells with glutamine starvation in conjunction with two different concentrations of cisplatin or etoposide for 18 h. Importantly, we found a strong enhancement of the apoptotic effect after glutamine starvation plus cisplatin, while no effect was seen with the glutamine starvation plus etoposide treatment (Fig. 5A).

Next, we evaluated the impact on cell proliferation as assessed by EdU incorporation in lCb-1299 primary cells treated with glutamine starvation or compound 968 with or without cisplatin for 18 h. Compound 968 is a cell-permeable inhibitor of GLS-2 that mimics a condition of glutamine starvation. We observed that glutamine starvation or compound 968 alone induced 25% or 30% inhibition in cell proliferation, respectively (Fig. 5B; Supplemental Fig. S5A). Importantly, the combinations of glutamine starvation plus cisplatin or compound 968 plus cisplatin induced a strong synergic effect of 45%–80% inhibition in cell proliferation (Fig. 5B; Supplemental Fig. S5A).

To assess whether p73 was mediating the apoptotic response upon glutamine starvation, we performed a stable p73 knockdown (p73sKD*3 and p73sKD*5). A robust apoptosis was observed in DAOY cells after glutamine starvation or glutamine starvation plus cisplatin after 30 h of treatment (Fig. 5C). Importantly, no effect was observed under glutamine starvation in both p73sKD cells, with only negligible apoptosis observed under glutamine starvation plus cisplatin in both p73KD cells. Furthermore, a strong reduction in ATP levels was observed in control DAOY cells after glutamine starvation or glutamine starvation plus cisplatin, but the effect was abolished in DAOY p73sKD (Fig. 5D). These data support the conclusion that p73 is an important element to mediate apoptosis in MB cells under glutamine starvation.

To confirm the “glutamine addiction phenotype” in MB primary cells, we evaluated the cell number and ATP levels after treatment with glutamine starvation, cisplatin, glutamine starvation plus cisplatin, 6-diazo-5-oxo-L-norleucine (DON; a glutamine antagonist), or DON plus cisplatin in m137 and lCb-1299 human primary cells. We demonstrated a synergistic effect resulting in a reduction of cell number only when cells expressing p73 were treated with the combination of glutamine starvation plus cisplatin or DON plus cisplatin (Fig. 5E). Instead, a strong reduction of ATP levels was observed already under glutamine starvation or DON treatment (Supplemental Fig. S5B), in keeping with MB cells expressing p73 using glutamine as a primary source of energy.

These results confirm the glutamine addiction phenotype of MB cells expressing p73.

### Glutamine starvation induces ROS and DNA damage in MB cells expressing p73

**Figure 6.**
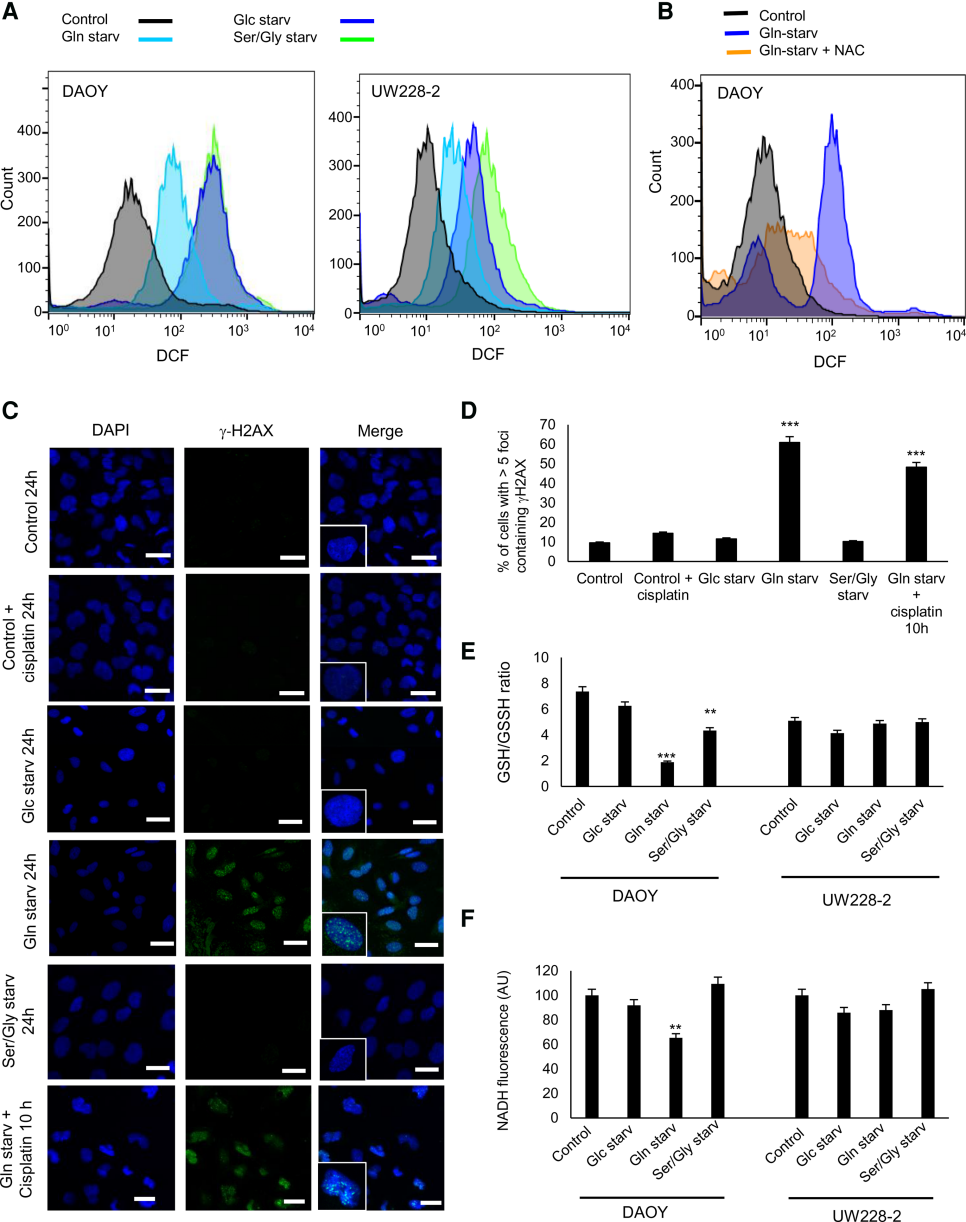
Glutamine starvation induces ROS and DNA damage in MB. (*A*) DAOY and UW228-2 cells were cultured for 12 h under normal medium, glucose starvation, glutamine starvation, and serine/glycine starvation. Cells were incubated with the H_2_DCFDA probe, an indicator for ROS. (*B*) DAOY cells were cultured for 12 h under normal medium, glutamine starvation, or glutamine starvation plus 0.1 µM NAC. Cells were incubated with H_2_DCFDA and analyzed by FACS. (*C*,*D*) DAOY cells were cultured under normal medium, glucose starvation, glutamine starvation, or serine/glycine starvation for 20 h. Also, DAOY cells were incubated with glutamine starvation plus cisplatin for 10 h. (*C*) Representative image of DAOY cells stained with the DNA damage marker phospho-Ser139 histone 2AX (γ-H_2_AX; green) and nuclear marker DAPI (blue). Bar, 20 µm. (*D*) Histogram shows mean fluorescence intensities of EdU in DAOY cells. Data are represented as mean ± SD. *n* = 3. (***) *P* < 0.0001. (*E*,*F*) DAOY and UW228-2 cells were cultured for 24 h under normal medium, glucose starvation, glutamine starvation, or serine/glycine starvation. (*E*) The reduced and oxidized glutathione ratio was measured by using the GSH/GSSG-Glo assay kit. (**) *P* < 0.001; (***) *P* < 0.0001. (*F*) Cells were collected, and NADH absorbance was determined at 340 nm by FACS analysis. (**) *P* < 0.001.

Nutrient deprivation induces ROS (Wu et al. 2013); therefore, we asked whether glucose, glutamine, or serine/glycine starvation could induce the accumulation of ROS with subsequent induction of DNA damage. We starved DAOY and UW228-2 cells of glucose, glutamine, or serine/glycine for 12 h followed by ROS assessment with the probe H_2_DCFDA. We observed that under the three starvation conditions, DAOY and UW228-2 cells underwent a redox imbalance reflected by increased ROS levels (Fig. 6A), an effect that was prevented by the addition of N-acetyl cysteine (NAC) (Fig. 6B; Supplemental Fig. S6A).

We next assessed whether the increased ROS levels induced DNA damage. DNA double-strand breaks (DSBs) were visualized by immunostaining for phospho-Ser139 histone 2AX (γ-H_2_AX) as nuclear foci at the sites of damage in DAOY cell nuclei 24 h after starvation. Confocal imaging of γ-H_2_AX foci indicated that glutamine starvation-induced foci that were more abundant and significantly larger in DAOY cells as compared with control cells (Fig. 6C,D). Similar findings were not observed after glucose or serine/glycine starvation treatment. Furthermore, the combination of glutamine starvation plus cisplatin induced an accumulation of foci in the nuclei at 10 h that was comparable with glutamine starvation alone after 24 h. These results suggest that cisplatin treatment induces a synergistic effect with glutamine starvation.

Next, we measured the redox status with the GSH/GSSG ratio as a marker for oxidative stress in DAOY and UW228-2 cells in control medium, glucose starvation, glutamine starvation, or serine/glycine starvation. We found that the levels of the GSH/GSSH ratio dropped significantly in DAOY cells under glutamine starvation, less so under serine/glycine starvation, and not significantly under glucose starvation (Fig. 6E). Importantly, no effect on the GSH/GSSH ratio was observed in UW228-2 cells.

To confirm this result, we measured NADH levels in these cells under the different starvation conditions because NADH levels are a well-recognized indicator for cellular metabolic homoeostasis. In fact, increased ROS levels and reduced NADH levels represent a status of metabolic imbalance (Li et al. 2016). We show a significant reduction of the NADH levels under glutamine starvation in DAOY cells, while no effect was observed in UW228-2 cells (Fig. 6F).

Together, these results suggest that MB cells that express p73 show a glutamine addiction phenotype at least in part by maintaining an oxidant detoxification capacity.

### Glutamine restriction diet reduces MB tumor growth

**Figure 7.**
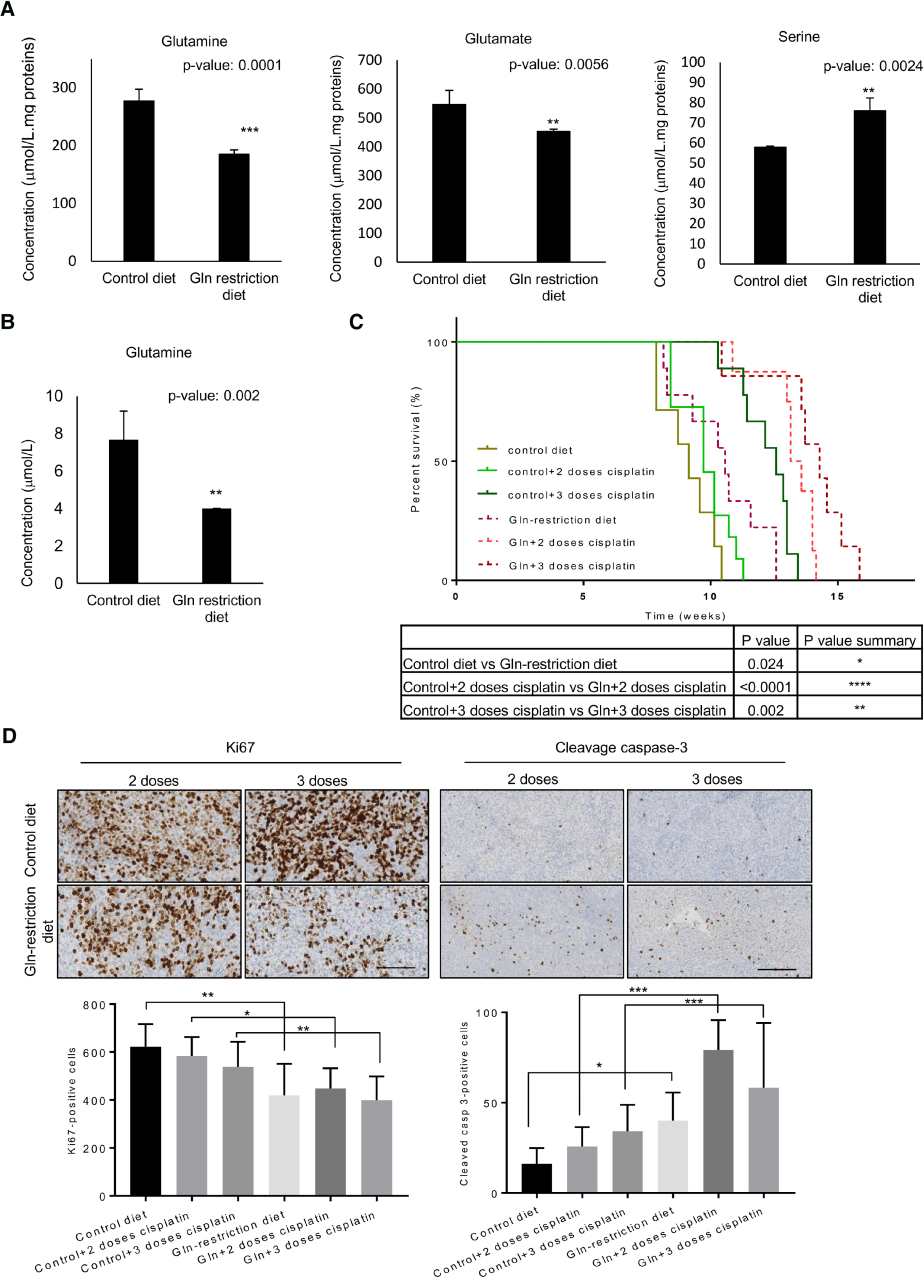
A glutamine restriction diet induces a significantly improved survival in an orthotopic MB xenograft model. (*A*,*B*) NOD-SCID mice were treated for 4 mo with a control diet or glutamine restriction diet. (*A*) Glutamine, glutamate, and serine levels were determined by ion-exchange chromatography in the cerebellum. (**) *P* < 0.001; (***) *P* < 0.0001. (*B*) Glutamine level was determined by ion-exchange chromatography in the CSF. Data are represented as mean ± SD. *n* = 5. (**) *P* < 0.001. (*C*,*D*) ICb-1299 human primary cells were injected into the cerebellum of newborn NOD-SCID mice, and mice were divided into control diet (*n* = 7), control diet + two doses of cisplatin (*n* = 11), control diet + three doses of cisplatin (*n* = 9), glutamine restriction diet (*n* = 9), glutamine restriction diet + two doses of cisplatin (*n* = 8), or glutamine restriction diet + three doses of cisplatin (*n* = 7). (*C*) The survival of the mice is plotted over time (log-rank test). (*) *P* < 0.01; (**) *P* < 0.001; (****) *P* < 0.00001. (*D*) Histology of the MB tumors under a control diet with two or three doses of cisplatin or under a glutamine restriction diet with two or three doses of cisplatin. Representative bright-field images are shown for Ki-67 and cleaved caspase-3. Quantification is shown as the mean of positive cells per high-power field. Bar, 50 µm. (*) *P* < 0.01; (**) *P* < 0.001; (***) *P* < 0.0001.

We next set out to validate our in vitro results in an in vivo mouse model of MB. First, we show that chronic depletion of nonessential amino acid glutamine (glutamine restriction diet) is well tolerated in vivo (Supplemental Fig. S7A). Subsequently, ion-exchange chromatography confirmed a significant drop in the levels of glutamine and glutamate, but not other amino acids, in the cerebellum and cerebrospinal fluid (CSF) of mice treated with a glutamine restriction diet (Fig. 7A,B; Supplemental Fig. S7B–D). Importantly, high levels of serine were observed in normal cerebellar tissue after a glutamine restriction diet (Fig. 7A), suggesting that normal cells could use serine metabolism for de novo ATP synthesis and survive under a glutamine restriction diet. Injection of ICb-1299 in the cerebellum of newborn NOD-SCID mice rapidly induced tumors with histological and immunohistochemical features of MB (Supplemental Fig. S7E). Animals fed with a glutamine restriction diet displayed a significant increase in survival time compared with those mice fed a control diet (Fig. 7C). Importantly, the combination of glutamine restriction diet with cisplatin (two and three doses) induced a significant synergic effect and extended the survival time (Fig. 7C). Indeed, we detected a significant reduction in cell proliferation (Ki67 staining) with a strong increase in apoptosis (cleaved caspase-3) in the animals fed a glutamine restriction diet while being treated with cisplatin as compared with control mice (Fig. 7D).

These results are in agreement with our in vitro data, in which glutamine starvation leads to reduced proliferation and increased apoptosis in synergism with cisplatin in MB cells expressing p73.

## Discussion

MB represents a heterogeneous group of brain tumors with distinct molecular and pathological features. Current treatments for MB include surgery, radiotherapy, and chemotherapy, which induce severe side effects in a substantial proportion of patients. We focused on elucidating the metabolic vulnerabilities of MB for the development of a new therapeutic strategy to minimize the adverse effects of the current therapy.

We show here that TAp73α is overexpressed in a proportion of MBs, including aggressive subgroups, a finding that confirms and further extends the original description of p73 protein overexpression in MB (Zitterbart et al. 2007).

Numerous evidence suggests that p73 has a well-defined role in cell metabolism and brain development (Agostini et al. 2016). However, because of the existence of numerous splice variants as well as the regulation of protein stability by proteasomal degradation and microRNA, its precise effect on cellular metabolism is currently ill defined.

We set out to assess whether p73 regulates any aspect of cell metabolism in human MB and characterize the relationship between p73 status and the sensitivity to glutamine restriction treatment. Therefore, we silenced p73 in MB cell lines and patient-derived MB cells and assessed metabolic changes by genome-wide transcriptome and global metabolite profiling. The global metabolite profiling is a novel and powerful approach to gain a comprehensive analysis of small endogenous metabolites (Nicholson et al. 1999). Such data can be used to form a hypothesis describing possible metabolic alternations due to perturbation of the cellular system (Haggblad Sahlberg et al. 2017). Overall, our study shows for the first time that TAp73 is essential for accurate mitochondrial bioenergetics at least in part by modulating GLS-2 expression, in agreement with previous data in a nonneoplastic context (Velletri et al. 2013).

The role of GLS-2 in tumorigenesis is context-specific and regulated by factors that are still incompletely characterized (Hensley et al. 2013). GLS-2 is a mitochondrial protein that hydrolyzes glutamine to glutamate. Following this, glutamate is used in the cells as a substrate in the TCA cycle or for glutathione synthesis. We show that p73KD induced growth inhibition in MB and is directly linked to reduced mitochondria respiration rates and glycolytic capacity. Notably, our data suggest that MB cells expressing p73 may be more dependent on mitochondrial respiration for ATP production. Taken together, these data suggest that TAp73 plays an essential role in the maintenance of MB cell energy.

Cells regulate energy levels through AMPK, which acts as a sensor of the ATP/ADP ratio (Hardie et al. 2012). AMPK activation induces metabolic changes leading to ATP renewal with inhibition of ATP consumption (Wu et al. 2013). The changes in ATP levels trigger the activation of the energetic stress responses (Mihaylova and Shaw 2011). The function of AMPK is partly mediated by mTOR, which lies at the heart of a nutrient-sensing signaling network that controls cellular metabolism (Liu et al. 2012). After p73KD, we observed a strong activation of AMPK with a robust inhibition of mTOR, suggesting that p73 maintains the activation of the mTOR pathway through modulation of AMPK activity.

We demonstrate that p73KD induces a robust reprograming of metabolic processes, including de novo glucose synthesis, also called gluconeogenesis. This process allows cells to synthetize serine/glycine and pentose phosphate pathway components from glycolytic intermediates. The role of serine in one-carbon metabolism to support the methionine cycle is well documented (Meiser et al. 2016) and was also implicated recently in de novo ATP synthesis (Maddocks et al. 2016). After p73KD, we observed enhanced rates of de novo purine synthesis and nucleoside, which may hint at an enhanced contribution to RNA and DNA synthesis. However, because p73KD induces inhibition of cell proliferation, it is most likely that the cells are using the serine/glycine pathway for ATP generation after p73 silencing, as demonstrated previously in colon cancer (Maddocks et al. 2016).

Over the past 40 years, it has become increasingly clear that tumor cells exhibit specific amino acid dependency, and cells from different tumors were shown to die quickly in vitro following arginine, cysteine, or glutamine deprivation, while normal cells survived (Liu et al. 2015). p53-deficient tumors are vulnerable to serine/glycine starvation (Maddocks et al. 2013), although current therapies do not fully exploit this major difference between cancer cells and nonneoplastic cells.

Here, we demonstrate that MB cells expressing p73 are “glutamine-addicted.” Glutamine plays an important role in supporting tumor growth, as it is essential for the synthesis of glutathione, an important mitochondrial antioxidant (Amores-Sanchez and Medina 1999). Glutathione is capable of preventing damage of different cellular components caused by ROS, such as free radicals, peroxides, or lipid peroxides (Mari et al. 2009). MB growth in vitro is inhibited in p73-expressing cells under glutamine starvation and is associated with an abrupt depletion of intracellular ATP. Importantly, MB cells under glutamine starvation show DNA damage even though all three starvation conditions induce overproduction of ROS. A wealth of literature supports the role of p73 in inducing apoptosis after DNA damage (Allocati et al. 2012). We show that only glutamine starvation induces TAp73 stabilization. Indeed, the combination of glutamine starvation with cisplatin (a chemotherapy agent of choice in MB therapy) induced a dramatic apoptotic response, possibly because of a mitochondria ROS response known to be triggered by cisplatin (Marullo et al. 2013). In keeping with this interpretation, treatment with etoposide did not elicit a similar effect. We validated p73's role in inducing apoptosis following glutamine starvation through the use of a stable p73 knockout. Crucially, these cells became refractory to glutamine starvation. This suggests that any drug inducing p73 stabilization or DNA damage will have a synergistic effect with glutamine starvation.

To validate these results in an in vivo mouse model of MB, we generated orthotropic xenografts of patient-derived MB cells expressing p73 in NOD-SCID mice. Kaplan-Meier analyses showed that a glutamine restriction diet induces a significant increase in the overall survival rate of the xenografted mice, with a significant reduction in cell proliferation (Ki67) and increase in apoptosis (cleavage caspase-3). Importantly, we observed a synergic effect between a glutamine restriction diet and cisplatin treatment, raising the possibility that glutamine restriction diets could be used as an adjuvant treatment for p73-expressing MB.

In summary, we show that p73 supports mitochondria respiration in MB via regulation of glutamine metabolism. Importantly, we validate the susceptibility of p73-expressing MB to a glutamine restriction diet in a xenograft model. The findings presented here support the notion that p73 is a marker of glutamine addiction in MB tumors and raise the possibility that a glutamine restriction diet could be implemented to maximize MB growth control while minimizing treatment toxicity.

## Material and methods

### LC-HRMS-based metabolite profiling

The analysis was performed using an Acquity UPLC I-class system from Waters coupled to a G2S Synapt Q-TOF equipped with an electrospray ionization (ESI) source (Waters). The sample separation was performed on a HILIC-Amide column (1.7-µm inner diameter, 2.1 × 50 mm) from Waters, and the column temperature was kept at 40°C.

### Extracellular amino acid measurement

The medium and cerebellar extracts were centrifuged, and the supernatant was analyzed. For CFS, at least 1 µL of CFS per animal was extracted. Next, 1 µL of CSF was diluted to 500 µL with deionized water and analyzed. Analysis was performed by ion-exchange chromatography, post-column derivatization with ninhydrin, and photometric detection.

### Extracellular glucose and lactate measurement

Media from DAOY control and sip73 cells were collected at different time points. The medium was centrifuged, and the supernatant was analyzed with an AccutrendPlus system (Roche) according to the manufacturer's instructions.

### Measurement of intracellular ROS

The DAOY and UW228-2 cells were incubated with 5 µM C-DCDHF-DA-AM (Invitrogen) for 30 min. ROS fluorescence (emission ∼530 nm) was measured by a 200-msec exposure (excitation ∼480 nm).

### Xenograft mouse models

All procedures had Home Office approval (Animals Scientific Procedures Act 1986, PPL:70/7275). NOD-SCID mice were divided randomly into two groups (control diet or glutamine restriction diet), each group consisting of 10 mice.

### Statistical analysis

All results are expressed as mean values ±SD or ±SEM of at least three independent experiments. The unpaired Student's *t*-test and analysis of variance (one-way ANOVA) were used to assess significant differences between results. *P*-values of <0.05 were considered statistically significant.

## Supplementary Material

Supplemental Material
