# Supplementary material for: TAp73 is a marker of glutamine addiction in medulloblastoma
Source: Genes Dev. 2017 Sep 1;31(17):1738–53. doi: 10.1101/gad.302349.117 (PMC5666673; doi:10.1101/gad.302349.117)
Supplement: Supplemental Material [file supp_31_17_1738__index.html]

TAp73 is a marker of glutamine addiction in medulloblastoma — Supplemental Material 

# TAp73 is a marker of glutamine addiction in medulloblastoma

## Supplemental Material

- Supplemental\_Fig7.pdf
- Supplemental\_Legends.pdf
- Supplemental\_Fig1.pdf
- Supplemental\_Fig4.pdf
- Supplemental\_Fig2.pdf
- Supplemental\_Fig5.pdf
- Supplemental\_Fig3.pdf
- Supplemental\_Fig6.pdf
