# Supplementary material for: TAp73 is a marker of glutamine addiction in medulloblastoma
Source: Genes Dev. 2017 Sep 1;31(17):1738–53. doi: 10.1101/gad.302349.117 (PMC5666673; doi:10.1101/gad.302349.117)

NiklisonChirou_FigS7

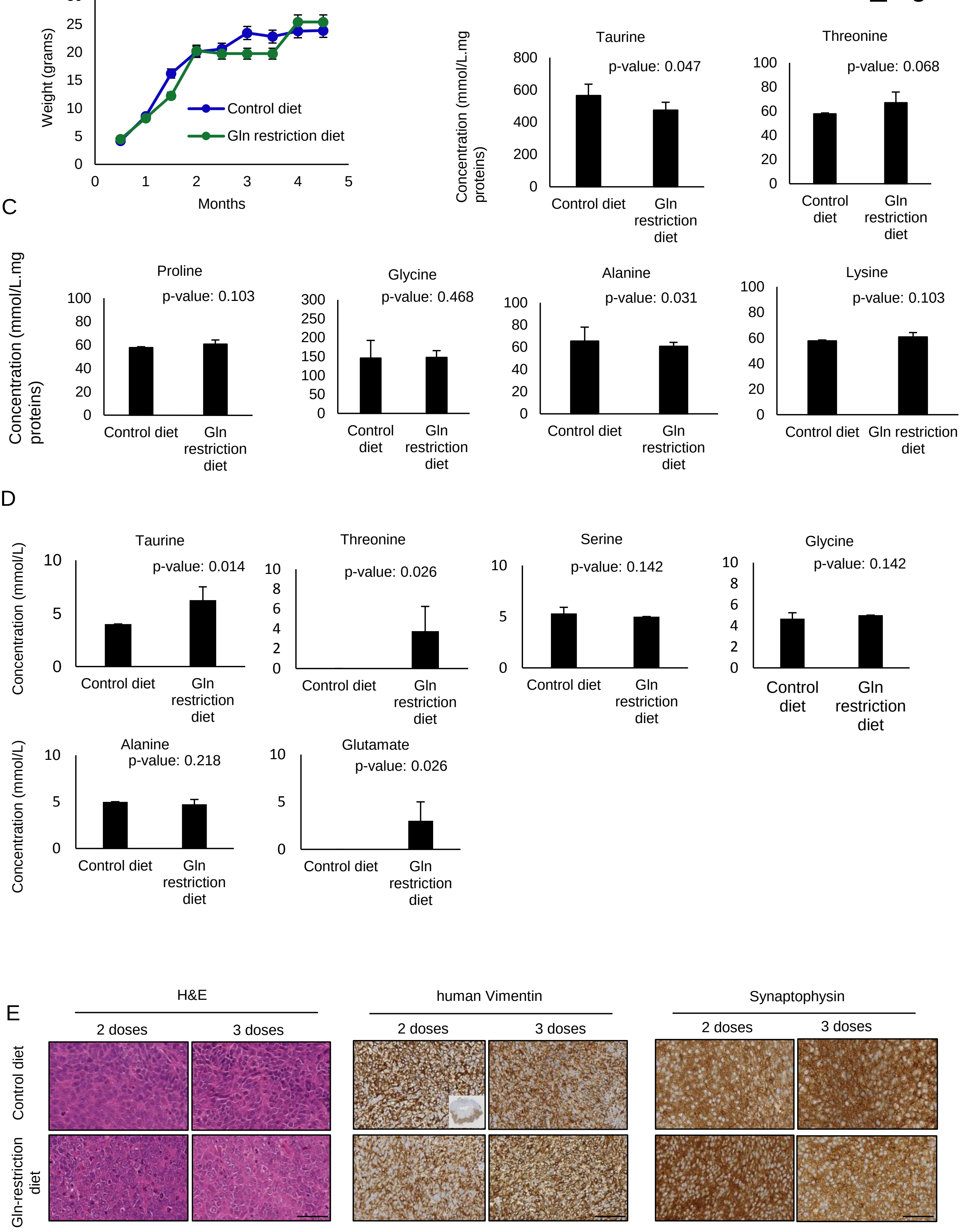

Supplement: Supplemental Material [file supp_gad.302349.117_Supplemental_Fig7.pdf]
