# Supplementary material for: TAp73 is a marker of glutamine addiction in medulloblastoma
Source: Genes Dev. 2017 Sep 1;31(17):1738–53. doi: 10.1101/gad.302349.117 (PMC5666673; doi:10.1101/gad.302349.117)
Supplement: Supplemental Material [file supp_gad.302349.117_Supplemental_Legends.pdf]

Supplemental Figure Legends

Figure S1. p73 is overexpressed in medulloblastoma and regulates GLS-2 expression.

A) Box plot representation for TAp73β, ΔNp73α and ΔNp73β expression levels (FPKM: fragments per kilobase of transcript per million mapped reads) across a cohort of 240 primary MB profiled on Affymetrix 1.1ST gene arrays. Patient number in each group are WNT: 28, SHH: 58, G3: 59, G4: 95 and CB: 3. CB: normal human childhood cerebellum.

B) RT-PCR was performed for TAp73 and ΔNp73 genes in DAOY cells. (n=3). Data are represented as mean ± SD. Values significantly lower than the control (***P<0.0001) are indicated.

C) DAOY, m137, ICb-1299, m692 and UW228-2 cells were collected in RIPA buffer and subjected to western blot analysis for the detection of p73. GAPDH was used as a loading control.

D, E, F) RT-PCR was performed for TAp73 (D), GLS-2 (E) and NGFR (F) genes in DAOY, UW228-2, ICb-1299, m137 and m692 cells (n=3). Data are represented as mean ± SD. Values significantly lower or higher than the control (***P<0.0001) are indicated.

G) DAOY cells were transient transfected with scramble or two siRNA against p73 (sip73*1: 2671, sip73*2: 115666). After 48h samples were collected in RIPA buffer and subjected to western blot analysis for the detection of p73, p63 and p53 levels. GAPDH was used as a loading control.

H) DAOY, m137 and ICb-1299 cells were transiently transfected with scramble or sip73*2. After 24h samples were collected and RT-PCR was performed for TAp73 gene (n=3). Data are represented as mean ± SD. Values significantly lower than the control (**P<0.001, ***P<0.0001) are indicated.

I) Gene expression analysis after silencing p73 in DAOY cells. DAOY cells were transiently transfected with scramble or sip73 (sip73*1: 2671) for 48h. Heat map representation of the most up or downregulated genes after p73 silencing. Genes involved in stress pathways were plotted. Genes were identified using MSigDB and plotted to highlight differences in expression of 3 z-scores or greater between the two groups (red = relative upregulation, blue = relative downregulation).

J) RNA-seq expression for GLS-2 analysis after silencing p73 in DAOY cells. DAOY cells were transiently transfected with scramble or sip73*1 for 48h.

K) Validation of the gene expression array by RT-PCR. DAOY cells was transiently transfected with scramble or sip73*1 for 0, 10, 24 and 48h. RNA was extracted and RT-PCR was performed for TAp73, GADD45B, GLS-2, ASL, SFN, PFKM, ACO2, NOS2, PhKA1, NBN, ENO2, AKT2, SDHA, CS and RAD50 genes. (n=3). Data are represented as mean ± SD.

Figure S2. p73KD induces mitochondria defects and activation of AMPK signalling

A) DAOY cells were transiently transfected with scramble or siRNA against GLS-2 (siGLS-2*1: s25941). After 24h samples were collected in RIPA buffer and subjected to western blot analysis for the detection of GLS-2 levels. Vinculin was used as a loading control.

B-C) Oxygen consumption rates (OCR) kinetic curve for m137 and ICb-1299 scramble and sip73 (sip73*1: 2671, sip73*2: 115666) cells. Kinetic response to oligomycin (2µM) to determine ATP coupled respiration, FCCP (0.3µM) to establish maximal respiratory capacity and rotenone (2µM)/Antimycin A (1µM) to define mitochondrial respiration. m137 (B) and ICb-1299 (C) cells were plated at 20,000 cells/well in XF-24 cell culture plates. OCR±SEM were recorded to construct bioenergetics profiles. A minimum of five different samples were analysed for each group. ***P<0.0001 (unpaired, two-sided t-test). A representative experimental dataset is shown.

D-E-F) DAOY cells were infected with an empty vector and with two different shRNA (p73sKD*3 and p73sKD*5). Cells were selected with puromycin.

D) Cells were collected in RIPA buffer and subjected to western blot analysis for the detection of p73. GAPDH was used as a loading control.

E) Control, p73sKD*3 and p73sKD*5 cells were transiently transfect with scramble or FLAG-GLS-2 expression vector. After 72h samples were collected in RIPA buffer and subjected to western blot analysis for the detection of the Flag epitope. Vinculin was used as a loading control.

F) Representative images of p73sKD*5 cells alone or after transfection with a FLAG-GLS-2 expressing vector for 72h. Cells were labeled with the mitochondrial marker MitoTracker® green (green) and staining with antibody against FLAG epitope (red) and nuclear marker DAPI (blue). A representative micrograph is shown. Magnification 63×. Size bar=20μm.

G) Oxygen consumption rates (OCR) kinetic curve for DAOY control, p73sKD*5 and p73sKD*5+GLS-2. Kinetic response to oligomycin (2μM) to determine ATP coupled respiration, to FCCP (0.3μM) to establish maximal respiratory capacity and to rotenone (2μM)/Antimycin A (1μM) to define mitochondrial respiration. Cells were plated at 20,000 cells/well in XF-24 cell culture plates. OCR±SEM were recorded to construct bioenergetics profiles. A minimum of five different samples were analysed for each group. ***P<0.0001 (unpaired, two-sided t-test). A representative experimental dataset is shown.

H) DAOY cells were transiently transfected with scramble or two siRNA against p73 (sip73*1: 2671, sip73*2: 115666) for 24h and then labeled with DiOC6(3) a marker of mitochondrial membrane potential. Cells were analysed by flow cytometry and the geometric means were recorded. (n=3). Data are represented as geometric means ± SD. Values significantly higher than the control (***P < 0.0001) are indicated.

I) ATP levels was measured in m137, ICb-1299 and DAOY cells. m137 and ICb-1299 cells were transiently transfected with scramble or sip73*1 for 24h. DAOY cells were transfected with scramble or siGLS-2 (siGLS-2*1: s25941, siGLS-2*2: s223735) for 24h. The cells were collected and ATP levels were determined with CellTiter-Glo Luminescent Cell Viability Assay solution. Strong reduction in ATP content was observed after p73 or GLS-2 silencing. (n=3). Data were mean ± SD. Values significantly lower than the control (***P < 0.0001, **P < 0.001) are indicated.

J) Relative intensity of the newly synthetized proteins. Western blot bands were quantified by ImageJ. DAOY cells were transiently transfected with scramble or two different shRNA against p73. After 48h, DAOY cells were treated for 1h with puromycin (2.5mg/ml). Next, cells were collected and a western blot of lysed cells probed with a primary anti-puromycin antibody. Tubulin was used as a loading control.

Figure S3. p73 regulates metabolic pathways in MB

A, B) Kinetic curve for serine (A) and glycine (B) levels in the medium of DAOY scramble and after siRNA against p73 (sip73*1: 2671). The medium of DAOY scramble and sip73*1 was collected at 0, 6, 20, 30 and 40h and amino acid levels were quantified by ion-exchange chromatography (n=3).

C) Gluconeogenesis in cancer cells. The major substrates for gluconeogenesis (lactate, alanine and pyruvate) are highlighted as enclosed boxes. Pyruvate from the cytosol is transported across the inner mitochondrial membrane by the pyruvate transporter. Following reduction of oxaloacetate to malate the malate is transported to the cytosol by the malate transporter. In the cytosol the malate is oxidized to oxaloacetate and the oxaloacetate then feeds into the gluconeogenic pathway via conversion to phosphoenolpyruvate.

Figure S4. p73 is a marker of glutamine addiction in MB

A) Flow cytometric analyses of autophagic activity using LC3-II. DAOY and UW228-2 cells were cultured in control medium or under glucose, glutamine or serine/glycine starvation. The cells were treated for 24h and then an anti-LC3-II antibody (0.25μg) was added after fixation and permeabilization for 30 min at room temperature. Goat anti-rabbit IgG-AlexaFluor-546 (0.125mg) was used as a fluorescent tag. Data were analysed in FlowJo.

B) Western blot for p73 levels in DAOY cell after Gln-starvation plus MG132. DAOY cells were cultured for 4, 6 and 8 hours under normal medium or glutamine (Gln starv) starvation condition alone or with 10μM MG132. Next, cells were collected and a western blot of lysed cells was performed. The primary antibody probe was anti-p73 antibody. GAPDH was used as a loading control.

C) UW228-2 cells were cultured for 24h under normal medium, glucose (Glc starv), glutamine (Gln starv) and serine/glycine (Ser/Gly starv) starvation condition. Apoptosis was measured with Annexin V-FITC and PI for flow cytometry analyses. A minimum of 3 different samples were analysed for each group. A representative experimental data is shown. Data were analysed in FlowJo.

D-E) Glucose uptake and lactate production in control medium or after Gln-starvation condition. DAOY cells were treated or not with Gln-starvation medium, samples were collected at 0, 6, 20, 30 and 40 hours and glucose (D) or

lactate (E) was determined, (n=3). Data are represented as mean ± SD. Values significantly lower than the control (***P<0.0001) are indicated.

F-G) Serine and glycine levels in control medium or after Gln-starvation condition. DAOY cells were treated or not with Gln-starvation medium, samples were collected at 0, 6, 20, 30 and 40 hours and serine (F) and glycine (G) was determine, (n=3). Data are represented as mean ± SD. Values significantly lower than the control (**P<0.001,***P<0.0001) are indicated.

Figure S5. Co-treatment of cisplatin plus glutamine starvation induces a synergic apoptotic effect in MB

A) Representative image of ICb-1299 cells under control medium, glutamine starvation (Gln starv) or 4μM compound 968, with or without cisplatin 16.6nM for 18h. Cells were stained with the proliferation marker Edu (green) and nuclear marker DAPI (blue). Size bar=20μm.

B) ATP levels was measured in DAOY, ICb-1299, m137 and UW228-2 cells. Cell were treated with 16.6nM cisplatin, glutamine starvation (Gln starv), Gln starv + 16.6nM cisplatin, 0.9mM DON or 0.9mM DON + 16.6nM cisplatin. The cells were collected and ATP levels were determined with CellTiter-Glo Luminescent Cell Viability Assay solution (Promega, Madison, WI, USA). Data are represented as mean ± SD, (n=3). Values significantly lower than the control (**P < 0.001, ***P < 0.0001) are indicated.

Figure S6. Glutamine restriction induces ROS and DNA damage in MB

A) Table summarizing the geometric means of H_2DCFDA fluoresce assessed by flow cytometry. DAOY cells were cultured for 12 or 24h under normal medium, glucose (Glc starv), glutamine (Gln starv) and serine/glycine (Ser/Gly starv) starvation condition. Or, we pre-incubate the cells for 1 hour with 0.1μM N-acetyl cysteine (NAC) and then, the cells were challenge for 12 hours with normal medium, glucose (Glc starv), glutamine (Gln starv) and serine/glycine (Ser/Gly starv) starvation condition. Cells were incubated for 30 min with probe H_2DCFDA, an indicator for reactive oxygen species (ROS) and immediately analyzing by flow cytometry. Data were analysed in FlowJo.

Figure S7. Glutamine restriction diet improves survival in an orthotopic MB xenograft model.

A) Males NOD-SCID mice were divided randomly into two groups: control diet (n=7) or Gln-restriction diet (n=8), and the weights were recorded at regular intervals. No difference was observed between the two different treatments.

B-C) Taurine, threonine, proline, glycine, alanine and lysine levels were determined in the cerebellum of mice under control diet and under Gln-restriction diet by ion-exchange chromatography. (n=3). Data are represented as mean ± SD.

D) Taurine, threonine, serine, glycine, alanine and glutamate levels were determined in the CSF obtained from mice under control diet and Gln-restriction diet by ion-exchange chromatography (n=3). Data are represented as mean ± SD.

E) Histology of MB tumors (H&E staining) under control diet while treated with cisplatin (2 or 3 doses) or under Gln-restriction diet while treated with cisplatin (2 or 3 doses). Widespread expression of synaptophysin is shown and human vimentin confirms their origin. H&E: haematoxylin and eosin. Scale bar is 250μm and 2mm for the inset.

Supplemental Experimental Procedures

Chemicals

Formic acid (LC-MS grade), ammonium formate (LC-MS grade) and methanol (LC-MS grade) were purchased from Sigma Aldrich (Steinheim, Germany). Acetonitrile (LC-MS grade) was obtained from Fischer Scientific (Zurich, Switzerland) and chloroform (analytical grade) was obtained from BDH Laboratory Supplies (Poole, England, UK). Leucine-encephalin was prepared and certified by ERA (Golden, CO, USA). The water was purified using a Milli-Q™ water system from MilliPore (Bedford, MA, USA).

Cell culture

Medulloblastoma cell lines DAOY, UW228-2 and the ICb-1299 primary MB cells were cultured at 37°C in humidified 5% CO_2 in DMEM + GlutaMAX medium (Gibco), supplemented with 10% v/v foetal bovine serum (FBS, Gibco) and penicillin/streptomycin (1 U/ml, Gibco), as previously described (Merve et al. 2014).

The m137 and m692 were grown as adherent monolayer culture in serum-free medium as described previously (Pollard et al. 2009). Briefly, cells were cultured at 37°C in 5% CO_2 in PLO/laminin-coated flasks to ensure adhesion in a serum-free medium Neurocult (Stemcell Technologies) containing 40 ng/ml epidermal growth factor (EGF; BD Biosciences) and 40 ng/ml fibroblast growth factor (FGF; BD Biosciences).

ICb-1299 primary MB cells were obtained from Dr Xiao-Nan Li, Baylor College of Medicine, Texas Children's Cancer Centre, USA and the m137 and m692 from Peter Dirks, The Hospital for Sick Children, University of Toronto, Canada. DAOY and UW228-2 cells were purchased from ATCC.

Starvation medium

Equal amounts of DAOY and UW228-2 cells were plated. After the cells were given time to attach, the medium was removed. The cells were then washed 5 times with PBS and the respective starvation mediums were added to the cells.

For serine/glycine starvation experiments, cells were cultured in complete medium: Minimum Essential Medium (MEM) no glutamine (Invitrogen), supplemented with MEM Vitamins (Invitrogen), 10% dialysed FBS (HyClone, Logan, UT, USA), L-Glutamine 2 mM and D-Glucose 25mM, as previously described (Amelio et al. 2014).

For glutamine starvation experiments, cells were cultured in complete medium: MEM no glutamine (Invitrogen), supplemented with MEM Vitamins (Invitrogen), 10% dialyzed FBS (HyClone), D-Glucose 25mM, Serine 0.4 mM, and Glycine 0.4 mM, as previously described (Willems et al. 2013).

For glucose starvation experiments, cells were cultured in complete medium: MEM no glutamine (Invitrogen), supplemented with MEM Vitamins (Invitrogen), 10% dialyzed FBS (HyClone), L-Glutamine 2 mM, Serine 0.4 mM, and Glycine 0.4 mM, as previously described (Graham et al. 2012).

Transient knockdown of p73 or GLS-2 with siRNA:

siRNA constructs targeting p73 (sip73*1: ID: 2671, sip73*2: ID: 115666), GLS-2 (siGLS-2*1: s25941, siGLS-2*2: s223735) and a non-targeting control siRNA (scramble) were purchased from Ambion. Cells were transfected with 10 pM siRNA with lipofectamine 3000 (Life Technologies) according to the supplier's protocol. Cells were collected a different time points after transfection. To assess knockdown efficiency, we used RT-PCR and western blot.

Transient re-expression of TAp73 or GLS-2:

Cells were transfected with human Flag-Gls2-expressing vector or mouse TAp73-GFP-expressing vector, kindly provided by Prof. Gerry Melino, with lipofectamine 3000 (Life Technologies) according to the supplier's protocol. Cells were collected a different time points after transfection.

Lentiviral gene transfer

Lentiviral plasmids shRNA vector containing a hairpin sequence targeting p73 (p73sKD*3: shTP73-1643 and p73sKD*5: shTP73-2076) or empty vector (SMC-002) were purchased from Sigma-Aldrich (Buchs, Switzerland) and kindly donate by Dr. Tschan. The shRNA-containing lentiviral vector was co-transfected with lentiviral packaging, using the calcium phosphate method into 293T cells. The medium was refreshed and the lentivirus was collected after 48 h after transfection, centrifuged briefly, and filtered through a 0.45-μM cellulose nitrate filter

(Millipore AG, Volketswil, Switzerland) and stored at −80°C. The infectious titre was determined using a BD FACS CANTO II flow cytometer analysing of GFP positive 293T cells (Di Foggia et al. 2014).

Lentiviral knockdown of p73 in DAOY cells

DAOY cells were seeded in multiple well plates and infected with p73 knockdown virus (10 MOI) or scramble (30 MOI) lentivirus in a proliferation medium. The medium was replaced on the second day and the cells were selected by puromycin 0.2 μg/ml. The transduction efficiency was analysed 96 hours after infection by RT-PCR and western blot.

Propidium Iodide Staining

Cell death was determined using the live cell PI exclusion test coupled with flow cytometry. In brief, cells (2×10^5) were harvested by trypsinization at the end of the experiments. Cells were washed once with phosphate buffer saline (PBS) and re-suspended in PBS containing 1 μg of PI/ml. The levels of PI incorporation were quantified by flow cytometry using a BD FACS CANTO II. Cell size was evaluated by forward-angle light scattering. PI-negative cells with normal size were considered to be live cells.

Extra-cellular amino acid measurement

Amino acid level was determined in Viapath, St Thomas' Hospital, UK. Briefly, medium from cells, cerebellum extraction or CSF, was collected. The medium and cerebellum extracts were centrifuged and the supernatant was analysed. For CFS, at list 1μL of CFS per animal was extracted. Follow, 1μL of CSF was diluted to 500μL with deionized water and analysed. Analysis was performed by ion-exchange chromatography, post column derivitisation with ninhydrin and photometric detection.

Extra-cellular glucose and lactate measurement

Glucose and lactate was determined in the Department of Clinical Biochemistry, Royal London Hospital, UK. Briefly, medium from DAOY control and sip73 cells was collected a different time point. The medium was centrifuged and the supernatant was analysed with an Accutrend® Plus System (Roche) according to the manufacturer's instructions.

Apoptosis determination

Apoptosis was quantified using the Annexin V-FITC Determination Kit (eBioscience) according to the manufacturer's instructions. Briefly, cells medium and the cells were collect in a FACS tube. Annexin V-FITC (25μg/ml) was then added to the cell suspension. The cells were then left to sit in the FACS tubes for 10 minutes in darkness at room temperature. Propidium iodide (1μg/ml) staining solution was then added and FACS was carried out. Experiments were carried out using a BD FACS CANTO II flow cytometer.

Cell Proliferation

Cell proliferation was quantified using the Click-iT EdU Imaging Kit (Invitrogen) according to the manufacturer's instructions.

Western blotting

Cells were collected and then sonicated for 5 min in RIPA buffer (50 mM Tris–HCl (pH 7.5), 150 NaCl, 1% NP-40, 0.25% sodium deoxycholate). Proteins were quantified using the Pierce™ Coomassie (Bradford) Protein Assay Kit and each sample was separated by SDS-PAGE. Proteins were transferred onto nitrocellulose membranes, which were blocked in 5% non-fat milk diluted in Tris buffered saline (TBS) containing 0.5% Tween 20 (TBS-T) for 30 min at room temperature. Primary antibodies were diluted in 5% milk in TBS-T and incubated at 4°C overnight.

We used rabbit p73 (1/1000, Bethyl), rabbit GLS-2 (1/1000, Abcam), rabbit ACC (1/1000, Cell Signalling), rabbit P-ACC (1/1000, Cell Signalling), rabbit S6K (1/1000, Cell Signalling), rabbit P-S6K (1/1000, Cell Signalling), rabbit eEF-2 (1/1000, Cell Signalling), rabbit P-eEF-2 (1/1000, Cell Signalling), rabbit puromycin (1/1000, Abcam) and mouse Flag-M2 (1/1000, Sigma). Mouse GAPDH (1/5000, Sigma Aldrich) and mouse Tubulin (1/5000, Santa Cruz) was used as a loading control.

The membranes were then washed three times for 10 min in TBS-T. Secondary antibodies anti-mouse (1/10000, Sigma-Aldrich) or anti-rabbit (1/10000, Sigma-Aldrich) were diluted in 5% non-fat milk in TBS-T and incubated for 1 hour at room temperature. The membranes were washed in TBS-T three times for 10 min. Then, membranes

were incubated in Lumi-Light Western blotting substrate (Roche Diagnostics, Indianapolis, IN). Membranes were exposed to film and developed.

Protein quantification

Protein quantification was perform using the Pierce™ Assay Kit according to the manufacturer's instructions. Samples were incubated at 37°C. The optimal wavelength to measure is 595nm which was carried out using a plate reader. We used the bovine serum albumin (BSA) solutions as a standard.

ATP measurement

Cell viability was quantified by CellTiter-Glo® Luminescent Cell Viability Assay according to the manufacturer's instructions. Detection is based on using the luciferase reaction to measure the amount of ATP from viable cells. The plate used was opaque to limit interference. The intensity of the emitted light due to the degradation of d-Luciferin and ATP by the enzyme Luciferase is proportional to the amount of free ATP present at that moment in the cells.

Real-time PCR

Total RNA was extracted from samples using Rneasy Mini Kit (Qiagen, Hilden, Germany) according to the manufacturer's instructions. RNA was quantified using spectrophotometry. DNA in the sample was then digested using Dnase 1 (Sigma) and RNA was converted to cDNA using RevertAid H minus First Strand cDNA Synthesis Kit (Thermo Fisher Scientific). For real-time PCR, we used SYBR green Supermix (Bio-Rad Laboratories, Hercules, CA, USA) according to the manufacturer's instructions.

Forward and reverse human primers used were (written 5' to 3'): TAp73 (fwd: GCACCTACTTTGACCTCCCC, rev:GCACTGCTGAGCAAATTGAAC); 18S (fwd:CAGCCACCGAGATTGAGCA, rev: TAGTAGCGACGGGCGGTGTG); GADD45B (fwd:CACCCTGATCCAGTCGTTCT, rev: CCCATTGGTTATTGCCTCTG); NOS2 (fwd:AGCTCAACAACAAATTCAGG, rev: ATCAATGTCATGAGCAAAGG); RAD50 (fwd:CTGAACGTGAGTTAAGCAAG, rev: GTATGATGGTTTAACTGCTCC); AKT2 (fwd:CACCATGAATGAGGTGAATAC, rev: CTACGGAGAAGTTGTTTAAGG); SFN (fwd:TCTGATCGTAGGAATTGAGG, rev: CACAGGGGAACTTTATTGAG); NBN (fwd:CAGGATTAAAGACAACAACTCC, rev: GTATCTGCTTGCTCTGATTC); ASL (fwd:CATCATGGAGAAGTTCAACG, rev: ATTGGAGTTCAGTTTGAAGG); ACO2 (fwd:GATCCACGAGACCAACC, rev: CAGGGTGAATCTTGTTGTTGTAG); PHKA1 (fwd:AGCTGCATATAAAACTGCTG, rev: TTCAACTCTGAGATCCCTTG); ENO2 (fwd:ATGTCCATAGAGAAGATCTGG, rev: GACACCTTTGCCTAAGTAAC); GLS-2 (fwd:CCATGGATATGGAACAGAAAG, rev: TTAACAACTTCGATGTGTCC); SDHA (fwd:TGGGAACAAGAGGGCATCTG, rev: CCACCACTGCATCAAATTCATG); PKM2 (fwd:ATTATTTGAGGAACTCCGCCGCCT, rev: ATTCCGGGTCACAGCAATGATGG); CS (fwd:GGTGGCATGAGAGGCATGAA, rev:TAGCCTTGGGTAGCAGTTTCT)

Measurement of Intracellular ROS

The DAOY and UW228-2 cells were incubated with 5 μM C-DCDHF-DA-AM (Invitrogen) for 30 min. C-DCDHF- DA is oxidized by ROS to dichlorofluorescein (DCF). ROS fluorescence (emission: ∼530 nm) was measured by a 200 ms-exposure (excitation: ∼480 nm) every 30 s using a BD FACS CANTO II. Recordings were started after a stable baseline was achieved.

Measurement of GSH/GSSG

DAOY and UW228-2 cells were plated and treated as indicated. Reduced and oxidized glutathione ratio was measured by using the GSH/GSSG-Glo assay kit (Promega) according to the manufacturer's protocol. Assay reagents were added directly to cells cultured in multi well plates. The GSH/GSSG-Glo™ Assay is a luminescence-based system for the detection and quantification of GSH/GSSG ratios in cultured cells. The luminescence intensity was measured using a Synergy HT Multi-Mode Microplate Reader.

Measurement of NADH

DAOY and UW228-2 cells were plated and treated as indicated. Cells were collect and suspend in 300uL of PBS. NADH can be measured by flow cytometry after excitation with a UV laser with a main emission peak at 470nm. Measurement of NADH levels can be used to monitor levels of oxidative phosphorylation.

Measurement of membrane potential

DAOY cells were plated and treated as indicated. Cells were collected and suspended in 300uL of PBS. 50nM of DiOC6(3) (Molecular Probes, Eugene, OR), was add for 30 min. Cells were centrifuged to remove the excess of the DiOC6(3). Samples were analyzed using a BD FACS CANTO II. Recordings were started after a stable baseline was achieved.

LC3-II measurement

DAOY and UW228-2 cells were plated and treated as indicated. Cells were incubated for 1 hour with 1:100 mouse anti-LC3-II (Abcam, ab51520) for 30 min and stained with 1:100 Goat anti-rabbit IgG-AlexaFluor-546 (Invitrogen) for 30 min. Samples were analyzed using a BD FACS CANTO II. Recordings were started after a stable baseline was achieved.

Newly synthetic proteins puromycin

DAOY cells were plated and treated as indicated. After 48h, DAOY cells were treated for 1h with puromycin 2.5mg/ml. DAOY cells were collect with RIPA buffer. The incorporation of puromycin in the newly synthetized proteins was assessed with an anti-puromycin antibody. Puromycin is known to bind easily to the C-terminal end of the growing peptide on ribosomes and thus stop further chain elongation.

Mitotracker green label

Coverslips were sterilized with 100% ethanol. p73sKD*5 DAOY cells alone or after transfection with a FLAG-GLS-2 were growth for 72h. Cells were label with 100nM MitoTracker® green (ThermoFisher, M7514) for 30 min at 37ºC. Next, the cells were fixed using 4% paraformaldehyde in PBS pH 7.4 for 10 min at room temperature. The samples were incubated for 10 min with PBS containing 0.1–0.25% Triton X-100. Afterwards, cells were washed in PBS three times for 5 minutes at a time. Cells were incubated with 10% Goat anti-serum and then left overnight at 4°C in the diluted antibody anti-Flag-M2 (1/250, Sigma) in 1% BSA in PBS-T in a humidified chamber. The solution were decanted and the cells washed three times in PBS leaving 5 minutes for each wash. Next, the cells were incubated with 0.1-1 μg/mL DAPI (DNA stain) for 5 min and then rinsed with PBS. The cells were incubated with the secondary antibody in (1/1000) 1% BSA for 1 h at room temperature in the dark. After decanting the secondary antibody solution and three washes with PBS for 5 minutes each in the dark, the coverslips were mounted on microscope slides with a drop of mounting medium. The coverslips were sealed with nail polish to prevent the cells from drying out and to ensure no movement when under the microscope. Images were taken using confocal microscopy and the resulting data was analysed using Image J.

Immunofluorescence

Coverslips were sterilized with 100% ethanol. DAOY and UW228-2 cells were then grown on glass coverslips. Then, the cells were grown for 24 hours in control medium or glucose, glutamine or serine/glycine starvation condition. Next, the cells were fixed using 4% paraformaldehyde in PBS pH 7.4 for 10 min at room temperature. The samples were incubated for 10 min with PBS containing 0.1–0.25% Triton X-100. Afterwards, cells were washed in PBS three times for 5 minutes at a time. Cells were incubated with 10% Goat anti-serum and then left overnight at 4°C in the diluted antibody anti-γ-H2AX (1/250) in 1% BSA in PBS-T in a humidified chamber. The solution were decanted and the cells washed three times in PBS leaving 5 minutes for each wash. Next, the cells were incubated with 0.1-1 μg/mL DAPI (DNA stain) for 5 min and then rinsed with PBS. The cells were incubated with the secondary antibody in (1/1000) 1% BSA for 1 h at room temperature in the dark. After decanting the secondary antibody solution and three washes with PBS for 5 minutes each in the dark, the coverslips were mounted on microscope slides with a drop of mounting medium. The coverslips were sealed with nail polish to prevent the cells from drying out and to ensure no movement when under the microscope. Images were taken using confocal microscopy and the resulting data was analysed using Image J.

MB gene expression profiling

RNA from DAOY scramble versus DAOY sip73 for 48h was extracted using the Qiagen RNeasy Micro Kit (Qiagen, UK) according to the manufacturer's descriptions. The concentration of RNA was confirmed using a spectrophotometer (Thermo-Scientific, Wilmington, DE, USA) at the wavelength ratios of A260/230 and A260/280 nm. Libraries were ribosome depleted, pair-end sequenced using the HiSeq 2000 system (Illumina Inc., San Diego, CA, USA) with a read length of 150 bp.

Hierarchical clustering was performed using Pearson distance and average linkage after gene-wise standardization. Differentially expressed probe sets were identified using ANOVA and p-values were adjusted for multiple testing using Benjamini-Hochberg correction in order to control the false discovery rate. An enrichment map was used to visualize results from pathway tests

The data obtained in this study was deposited into the international public repository Gene Expression Omnibus (GEO) database, where it has been assigned an ID number: GSE103029.

Xenografts mouse model

All procedures had Home Office approval (Animals Scientific Procedures Act 1986, PPL 70/7275). NOD-SCID P4-6 mice were anaesthetized according to standard procedure. Tumor cells (10^5 cells resuspended in 2µl sterile PBS) were injected into the right cerebellar hemisphere (2mm lateral and 2mm posterior to lambda, 2mm deep) with a 26 gauge Hamilton syringe needle (Merve et al. 2014). Mice were divided randomly into two groups (Control diet or Gln deficient diet), and each group consisted of 10 mice.

Control diet - 5BYA (TestDiet, International Product Supplies), ingredients: sucrose (20.0%), corn starch (36.4%), corn oil (5.0%), AIN 93M mineral mix (3.5%), AIN 93 vitamin mix (1.3%), maltodextrin (15%), sodium bicarbonate (1.0%), choline bitartrate (0.25%), amino acid premix (16.0%). Amino acid pre-mix: L-arginine-HCL (1.60%), L-cystine (0.64%), L-glutamine (1.60%), glycine (1.33%), L-histidine-HCL (0.80%), L-isoleucine (1.07%), L-leucine (1.60%), L-lysine-HCL (1.87%), L-methionine (0.80%), L-phenylalanine (1.07%), L-serine (1.33%), L-threonine (1.07%), L-tryptophan (0.27%), L-tyrosine (0.53%), L-valine (1.07%).

The Gln deficient diet – 5BYB (TestDiet, International Product Supplies), has the same basic formulation as the control diet, but the amino acid mix lacks Gln. Gln-free diet ingredients: sucrose (20.0%), corn starch (36.1%), corn oil (5.0%), AIN 93M mineral mix (3.5%), AIN 93 vitamin mix (1.3%), maltodextrin (15%), sodium bicarbonate (1.0%), choline bitartrate (0.25%), amino acid premix (16.0%). Amino acid pre-mix: L-arginine-HCL (2.0%), L-cystine (0.64%), glycine (1.4%), L-histidine-HCL (0.92%), L-isoleucine (1.23%), L-leucine (1.9%), L-lysine-HCL (2.1%), L-methionine (0.80%), L-phenylalanine (1.23%), L-serine (1.4%), L-threonine (1.23%), L-tryptophan (0.27%), L-tyrosine (0.61%), L-valine (1.23%).

The diets had equal calorific value and equal total amino acid content. Animals were housed in sterile IVC cages, monitored thrice weekly and killed humanely when developing neurological signs.

Histological examination and immunostaining

Mice were killed when developing neurological signs and brains were removed and placed in 10% formalin for 24 h and then transferred to PBS. Paraffin embedding, coronal sectioning of 3-µm and staining for hematoxylin and eosin, synaptophysin and human vimentin were performed by UCL IQPath (Institute of Neurology, London, UK). Immunostaining was done on Ventana Discovery XT instrument an automated staining machines (ROCHE, Burgess Hill, UK) following the manufacturer's guidelines, using horseradish-peroxidase-conjugated streptavidin complex and diaminobenzidine as a chromogen. The following antibodies were used for histological characterisation: synaptophysin (Invitrogen 080130, prediluted), human vimentin (Roche 790-2917, prediluted), Ki67 (Dako, prediluted) and cleaved caspase-3 (Novocastra, prediluted).

Image capturing and analysis

Histological slides were digitised on a LEICA SCN400 scanner (LEICA UK) at 40× magnification and 65% image compression setting, and images were stored on Slidepath Digital Image Hub (Leica Microsystems).

RNA-seq analysis

Paired-end sequencing ~90M reads was performed on a cohort of 240 subgrouped primary MB patients. Libraries were normalised by overall size. Reads mapping to genes of interest TAp73α, GLS-2 and p75NTR were counted and the relative abundance of genes/isoforms were calculated using cufflinks/cuffnorm and the GENCODE17 transcriptome library.

The normal human cerebellar (CB) data were taken from GEO: https://www.ncbi.nlm.nih.gov/geo/query/acc.cgi?acc=GSE78564.

XF assay medium

OCR base assay medium was prepared as follows. 8.3g/L Dulbecco's Modified Eagle's Medium (DMEM) powder (Sigma, catalog number D5030), 1.85g NaCl (Sigma S3014), 10mL GlutaMAx (200mM, GIBCO 35050-061), 15mg Phenol red (Sigma P5530), 10mL Sodium Pyruvate (100mM, Sigma S8636) and 2g Glucose (11.11mM) pH:7.4. Final volume 1L.

ECAR base assay medium was prepared as follows. 8.3g/L Dulbecco's Modified Eagle's Medium (DMEM) powder (Sigma, catalog number D5030), 1.85g NaCl (Sigma S3014), 10mL GlutaMAx (200mM, GIBCO 35050-

061), 15mg Phenol red (Sigma P5530) and 10mL Sodium Pyruvate (100mM, Sigma S8636) pH:7.4. Final volume 1L.

Pre-coated of the XF-24 microplate

XF-24 cell culture microplate was coated with 3.4 μg/mL BD Cell-Tak™ tissue adhesive solution (BD Bioscience 354240) was mixed with 8.4mg/ml sodium bicarbonate pH:8.0 and sodium hydroxide 40mg/ml according to manufacturer's instruction.

43μl of the Cell-Tak solution was added to each well of a XF-24 cell culture plate and incubated for 20 min a room temperature.

Measurements of oxygen consumption rates and extracellular acidification rates

Real-time measurements of oxygen consumption rates (OCR) and extracellular acidification rates (ECAR), were performed on a Seahorse XF-24 extracellular flux analyzer (Seahorse Biosciences) according to manufacturer's instructions and as previously described (Pike Winer and Wu 2014).

Briefly, 30.000 MB cells/well were attached to Seahorse XF-24 plates, which are pre-coated with Cell-TAK for metabolic analysis. After centrifugation of the microplate containing the cell suspension at 700×g for 5 min to facilitate cell attachment, cells were incubated at 37°C for at least 1h to allow attachment.

Drug was added automatically during measurement, after establishing baseline of oxygen consumption rates. We measured the OCR and ECAR in response to sequential treatment with the ATPase inhibitor oligomycin (2.5μM), the uncoupling agent FCCP (1μM) and the electron-transport-chain inhibitors rotenone (2μM) and antimycin A (1μM). Upon completion of an assay, cells were fix with 4% PFA and the nucleus were stain with DAPI. Follow the cells were image and count with IN Cell Analyzer 2000 for the purpose of normalization.

Cell growth and treatment for LC-HRMS

DAOY cells were cultured in a 10 cm dish at 37°C in humidified 5% CO_2 in DMEM + GlutaMAX medium (Gibco), supplemented with 10% v/v foetal bovine serum (FBS, Gibco) and penicillin/streptomycin (1 U/ml, Gibco), as previously described (Merve et al. 2014). For scrambled cells: DAOY cells were transfected with 10 pM of non-targeting control siRNA (scramble- Ambion) with lipofectamine 3000 (Life Technologies) to a density of 60%. Next day, the medium was changed and the cells were collected after 48h to a density of 90% (N=9). For silence p73 DAOY (sip73): DAOY cells were transfect with 10 pM siRNA construct targeting p73 (sip73*1: ID: 2671-Ambion) with lipofectamine 3000 (Life Technologies) to a density of 80-85%. Next day, the medium was changed and the cells are collected after 48h to a density of 90% (N=9). We include 3 biological replicate with 3 technical replicates for each condition.

Quenching and harvesting for LC-HRMS

After 48 hours of transfection the cells were washed three times with ice-cold PBS (3X 5mL) and the cells were collected using a cell scraper. 3 mL of ice cold H_2O (sterile, Milli Q) was added to each samples. The suspension was transfer into a 15 mL polypropylene tube. Next, the cells were snap freeze in liquid N_2, and store on ice for 5 minutes. Follow, the cells were lyse by two cycle of freeze-thaw, thaw samples at 37ºC for 10 min using a water bath and freeze using liquid N_2. Subsequently, the cells were sonicate in a "Bioruptor sonicator" for 30s at medium power. 50μL of samples were taken for protein normalization. The samples are store at -80ºC.

Metabolite extraction for LC-HRMS

The samples were thawed at room temperature and subjected to centrifugation for 17 min at 3000 rpm and 4°C. A quality control (QC) sample was created by pooling an equal volume from all samples. The QC sample was prepared as described below. The aqueous supernatants were transferred to fresh extraction tubes followed by addition of chloroform and methanol for the final proportion 2.85:4:4 water:methanol:chloroform (Viant 2007; Leon et al. 2013). The extraction tubes were gently vortexed and then stored at 8°C for 20 min prior to centrifugation for 20 min at 3000 rpm and 4°C. The aqueous phases were recovered and evaporated to dryness at 40°C under N2. All samples were stored at -80°C after evaporation. Prior to analysis the samples were reconstituted in acetonitrile:Milli-Q water 90:10

Metabolite profiling with LC-HRMS

The analysis was performed using an Acquity UPLC I-class system from Waters (Manchester, UK) coupled to a G2S Synapt Q-TOF equipped with an electro spray ionization (ESI) source (Waters). All systems were controlled using Masslynx version 4.1 (Waters). The sample separation was performed on a HILIC-Amide column (1.7 µm,

i.d. 2.1x50 mm) from Waters and the column temperature was kept at 40°C. The mobile phase consisted of 90:10 acetonitrile/water with 10 mM ammonium formate and 0.1% FA (A) and 50:50 acetonitrile/ water with 10 mM ammonium formate and 0.1% FA (B). A non-linear gradient elution profile from 100 % A to 100 % B was used. In detail; 100% A was kept for 0.5 min then decreased non-linearly (slope-factor 8 in MassLynx) over 12.5 min to 100% B, 100% B was held for 3 min followed by 7 min at 100 % A to re-equilibrate the column for a total run-time of 23 min. The flow-rate was set at 0.3 ml/min throughout all analyses and the injection volume was 5 µl. Detection was performed in resolution MSE-mode in both positive and negative ionization mode within the scan-range m/z 50-800. The capillary voltage was 1 kV and 2 kV in positive and negative ionization mode respectively and the cone voltage was set to 30 V and 25 V respectively. Source temperature was 120°C in both ionization modes and the desolvation temperature was 500°C and 450°C in positive and negative mode respectively. Nitrogen was used as desolvation and cone gas at the respective flow-rates 800 l/h and 50 l/h. Argon was used as collision gas and a collision energy ramp from 20 eV to 45 eV was used for MSE acquisition. Lock-mass correction was applied using a solution of Leucine-Enkephalin.

Prior to the analysis of study samples, the QC sample was analyzed repeatedly to ensure stable analytical conditions. All study samples were analyzed in triplicates and the run-order was randomized. In between the study samples, with regular intervals, the QC sample was analyzed in triplicates to monitor the analytical stability throughout the analysis.

Data processing

The raw data was converted to NetCDF files using the software DataBridge (Masslynx version 4.1, Waters). Peak detection and retention time alignment was performed using the R based software XCMS (Smith et al. 2006). The cent Wave function was used for peak detection and the function parameters were set as follows; the maximal deviation in m/z between scans was set to 8 ppm, the maximal and minimal peak width was set to 5 and 25 s respectively and the signal to noise ratio cutoff was set to 10. Retention time correction was performed using the "obiwarp" function. The resulting dataset was exported to Microsoft Excel and all features with a retention time less than 45 s were removed. The data was normalized using median fold change. After normalization, all features with coefficient of variance (CV) >30 % in the QC samples were excluded (Want et al. 2010; Dunn et al. 2011).

Multivariate and univariate data analysis

The reduced data sets from the analysis was analyzed by multivariate data analysis using SIMCA-P+ (version 14, Umetrics, Umeå, Sweden) computational software package. All data was pareto scaled prior to further data analysis. Principal Component Analysis (PCA) and Orthogonal Projection to Latent Structures- Discriminant Analysis (OPLS-DA) in combination with S-Plots were used in order to identify differentiating features between sample groups, DAOY scramble cells and p73 knockdown cells (Trygg et al. 2007).

Features with p-values greater than 0.4 and less than -0.4 (corresponding to up- or down regulated features) were selected and annotated. The obtained experimental m/z values were used to search against the Human Metabolome Database (HMDB) (Wishart et al. 2009; Wishart et al. 2013), METLIN (Sana et al. 2008) and in-house databases with a molecular weight difference tolerance of ± 0.020 Da. The plausible hits were matched against the raw data signal isotopic pattern, fragmentation (when reference spectra was available) as well as related adducts present at the same retention time in the raw data. When possible the annotated metabolites were identified by analysis of analytical standards in the same analytical setup as the study sample analysis.

Annotated features were subjected to univariate data analysis with student's t-test using Microsoft Excel. Metabolite levels were considered significantly altered if the p-value was <0.05. Fold changes with 95 % confidence intervals for the significantly altered metabolites were calculated using Fieller's theorem.

The data obtained in this study was deposited into the NIH Common Fund's Data Repository and Coordinating Center, http://www.metabolomicsworkbench.org, where it has been assigned a Metabolomics Workbench Project ID:1225.

Supplemental References

Amelio I, Markert EK, Rufini A, Antonov AV, Sayan BS, Tucci P, Agostini M, Mineo TC, Levine AJ, Melino G. 2014. p73 regulates serine biosynthesis in cancer. *Oncogene* **33**: 5039-5046.

Di Foggia V, Zhang X, Licastro D, Gerli MF, Phadke R, Muntoni F, Mourikis P, Tajbakhsh S, Ellis M, Greaves LC et al. 2014. Bmi1 enhances skeletal muscle regeneration through MT1-mediated oxidative stress protection in a mouse model of dystrophinopathy. *J Exp Med* **211**: 2617-2633.

Dunn WB, Broadhurst D, Begley P, Zelena E, Francis-McIntyre S, Anderson N, Brown M, Knowles JD, Halsall A, Haselden JN et al. 2011. Procedures for large-scale metabolic profiling of serum and plasma using gas chromatography and liquid chromatography coupled to mass spectrometry. *Nat Protoc* **6**: 1060-1083.

Graham NA, Tahmasian M, Kohli B, Komisopoulou E, Zhu M, Vivanco I, Teitell MA, Wu H, Ribas A, Lo RS et al. 2012. Glucose deprivation activates a metabolic and signaling amplification loop leading to cell death. *Mol Syst Biol* **8**: 589.

Leon Z, Garcia-Canaveras JC, Donato MT, Lahoz A. 2013. Mammalian cell metabolomics: experimental design and sample preparation. *Electrophoresis* **34**: 2762-2775.

Merve A, Dubuc AM, Zhang X, Remke M, Baxter PA, Li XN, Taylor MD, Marino S. 2014. Polycomb group gene BMI1 controls invasion of medulloblastoma cells and inhibits BMP-regulated cell adhesion. *Acta Neuropathol Commun* **2**: 10.

Pike Winer LS, Wu M. 2014. Rapid analysis of glycolytic and oxidative substrate flux of cancer cells in a microplate. *PLoS One* **9**: e109916.

Pollard SM, Yoshikawa K, Clarke ID, Danovi D, Stricker S, Russell R, Bayani J, Head R, Lee M, Bernstein M et al. 2009. Glioma stem cell lines expanded in adherent culture have tumor-specific phenotypes and are suitable for chemical and genetic screens. *Cell Stem Cell* **4**: 568-580.

Sana TR, Roark JC, Li X, Waddell K, Fischer SM. 2008. Molecular formula and METLIN Personal Metabolite Database matching applied to the identification of compounds generated by LC/TOF-MS. *J Biomol Tech* **19**: 258-266.

Trygg J, Holmes E, Lundstedt T. 2007. Chemometrics in metabonomics. *J Proteome Res* **6**: 469-479.

Viant MR. 2007. Revealing the metabolome of animal tissues using 1H nuclear magnetic resonance spectroscopy. *Methods Mol Biol* **358**: 229-246.

Want EJ, Wilson ID, Gika H, Theodoridis G, Plumb RS, Shockcor J, Holmes E, Nicholson JK. 2010. Global metabolic profiling procedures for urine using UPLC-MS. *Nat Protoc* **5**: 1005-1018.

Willems L, Jacque N, Jacquel A, Neveux N, Maciel TT, Lambert M, Schmitt A, Poulain L, Green AS, Uzunov M et al. 2013. Inhibiting glutamine uptake represents an attractive new strategy for treating acute myeloid leukemia. *Blood* **122**: 3521-3532.

Wishart DS, Jewison T, Guo AC, Wilson M, Knox C, Liu Y, Djoumbou Y, Mandal R, Aziat F, Dong E et al. 2013. HMDB 3.0--The Human Metabolome Database in 2013. *Nucleic Acids Res* **41**: D801-807.

Wishart DS, Knox C, Guo AC, Eisner R, Young N, Gautam B, Hau DD, Psychogios N, Dong E, Bouatra S et al. 2009. HMDB: a knowledgebase for the human metabolome. *Nucleic Acids Res* **37**: D603-610.
