# Supplementary material for: TAp73 is a marker of glutamine addiction in medulloblastoma
Source: Genes Dev. 2017 Sep 1;31(17):1738–53. doi: 10.1101/gad.302349.117 (PMC5666673; doi:10.1101/gad.302349.117)
Supplement: Supplemental Material [file supp_gad.302349.117_Supplemental_Fig1.pdf]

NiklisonChirou_FigS1

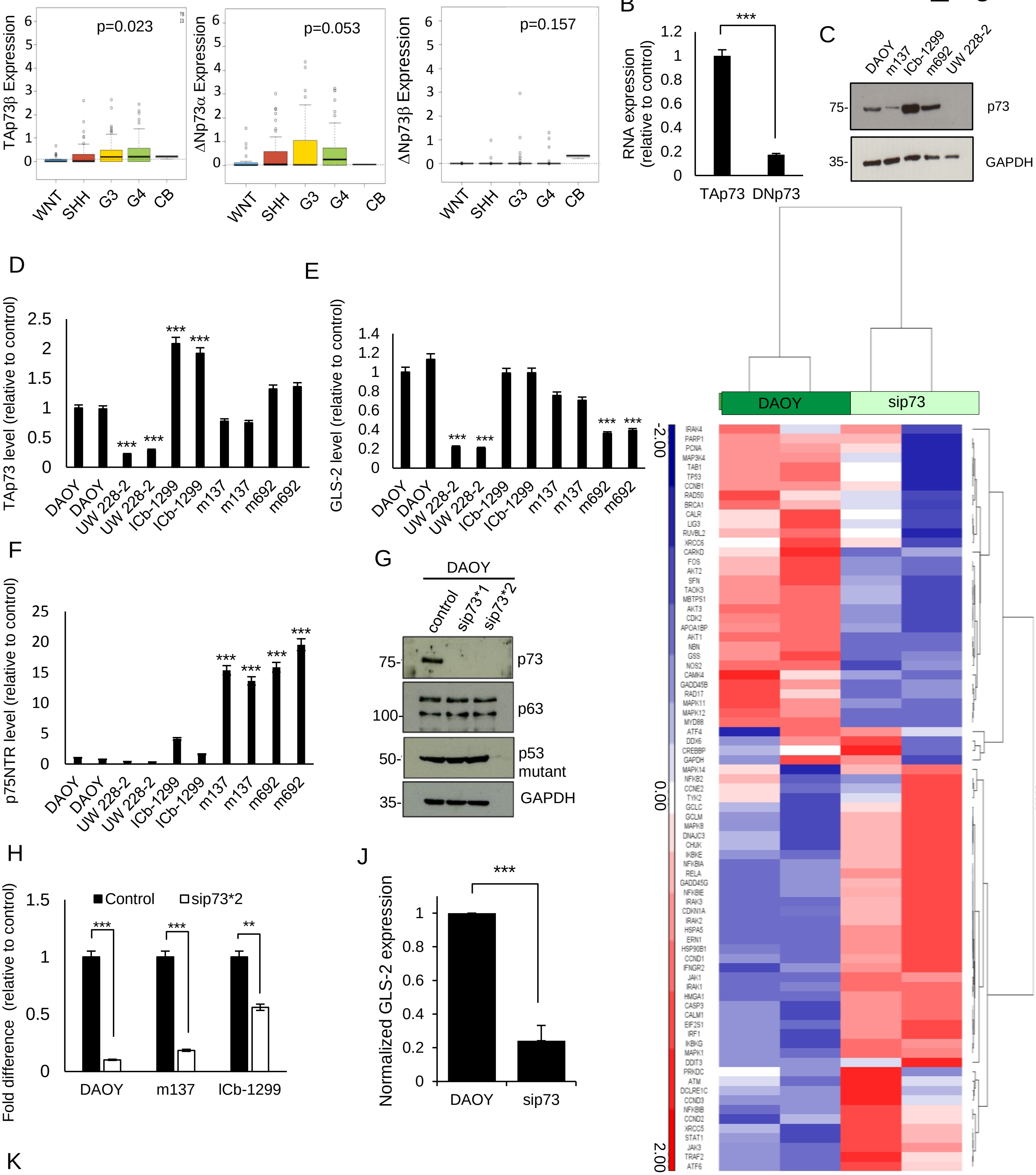

K

Fold difference (relative to control)															
Sample	TAp73	GADD45B	GLS-2	ASL	SFN	PFKM	ACO2	NOS2	PhKA1	NBN	ENO2	AKT2	SDHA	CS	RAD50
Control	1	1	1	1	1	1	1	1	1	1	1	1	1	1	1
10h	0.37	0.22	0.35	0.44	0.51	0.64	0.65	0.68	0.76	0.94	1.06	1.12	1.25	1.67	1.11
24h	0.54	0.29	0.35	0.29	0.90	0.27	0.64	1.16	0.90	0.65	0.76	0.78	1.05	1.07	1.42
48h	0.31	0.18	0.43	0.31	0.35	0.37	0.42	0.82	0.68	0.45	0.81	0.79	0.76	0.75	1.05
