# Supplementary figures and images for: TAp73 is a marker of glutamine addiction in medulloblastoma
Source: Genes Dev. 2017 Sep 1;31(17):1738–53. doi: 10.1101/gad.302349.117 (PMC5666673; doi:10.1101/gad.302349.117)

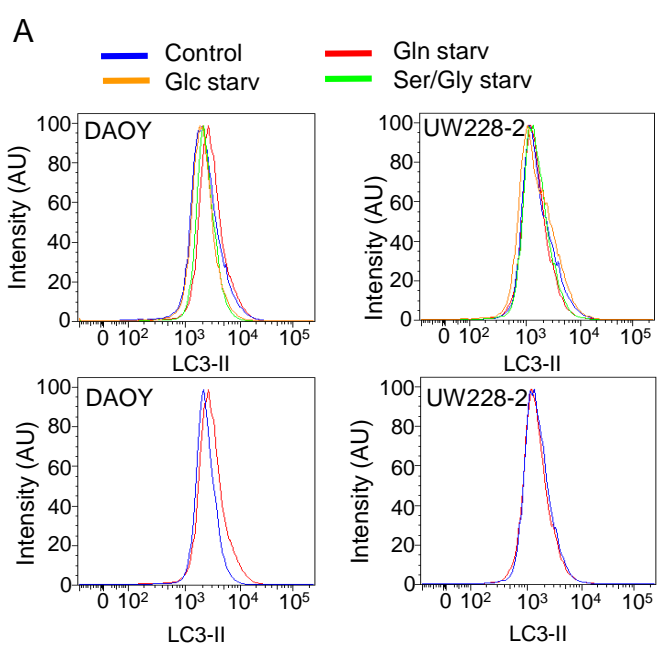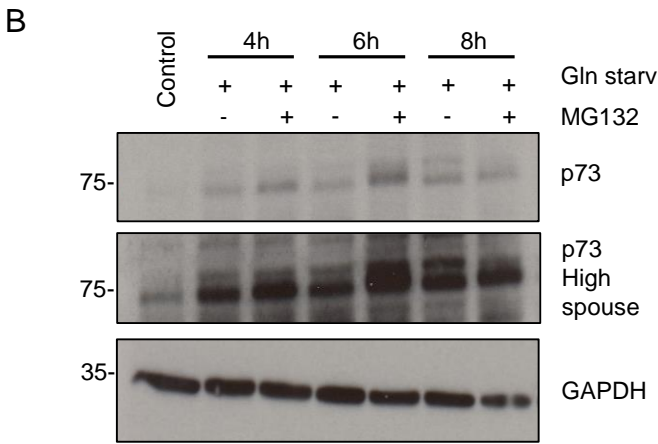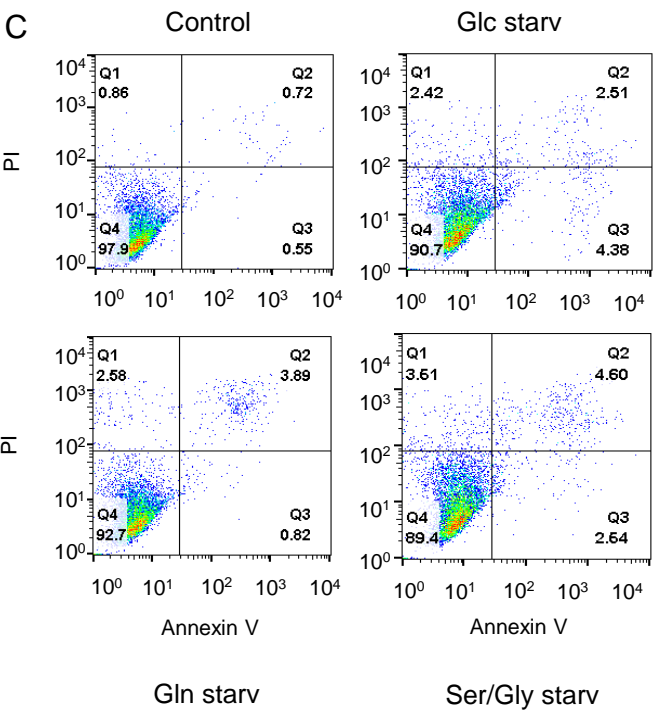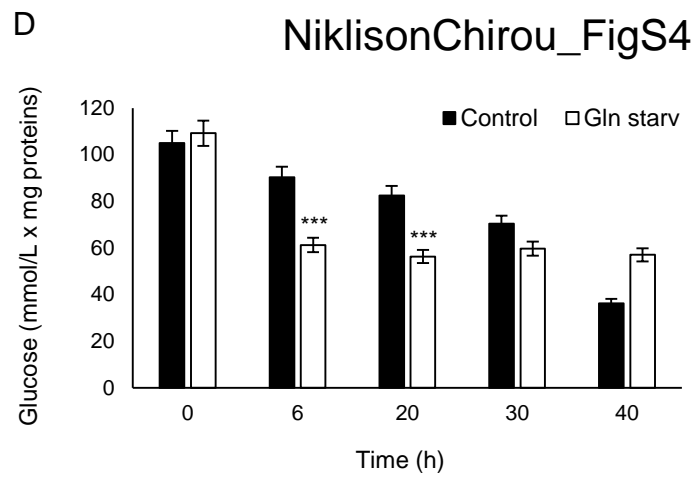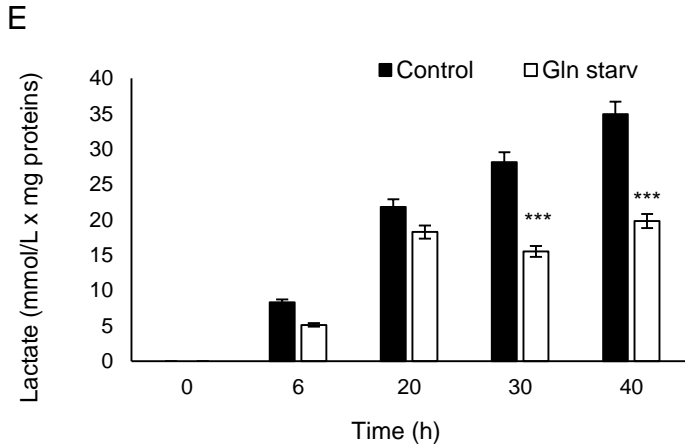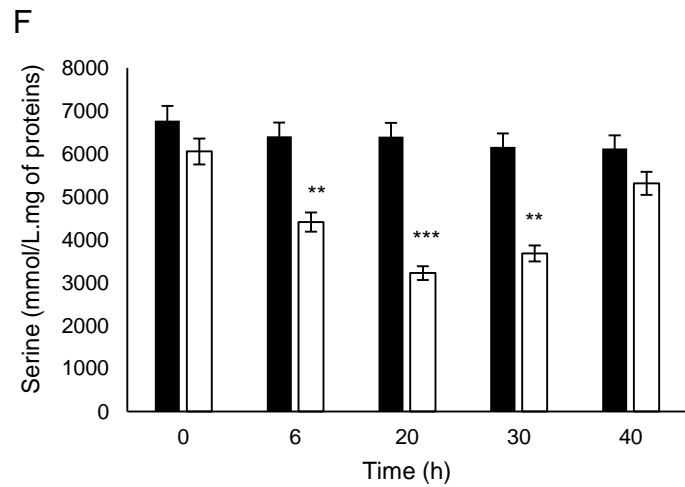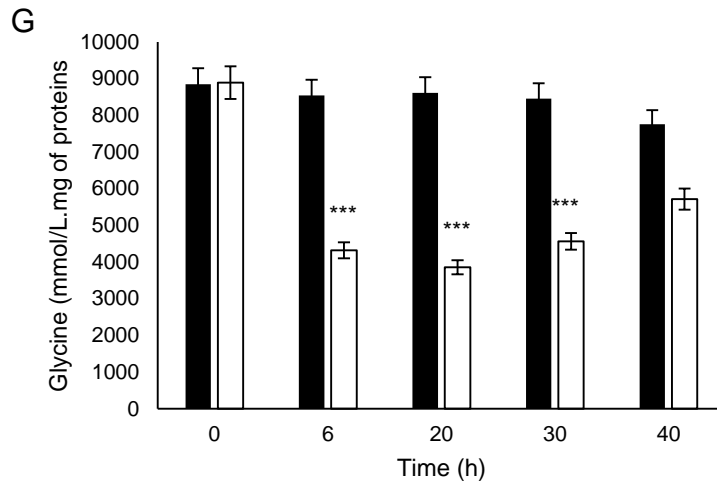

Supplement: Supplemental Material [file supp_gad.302349.117_Supplemental_Fig4.pdf]

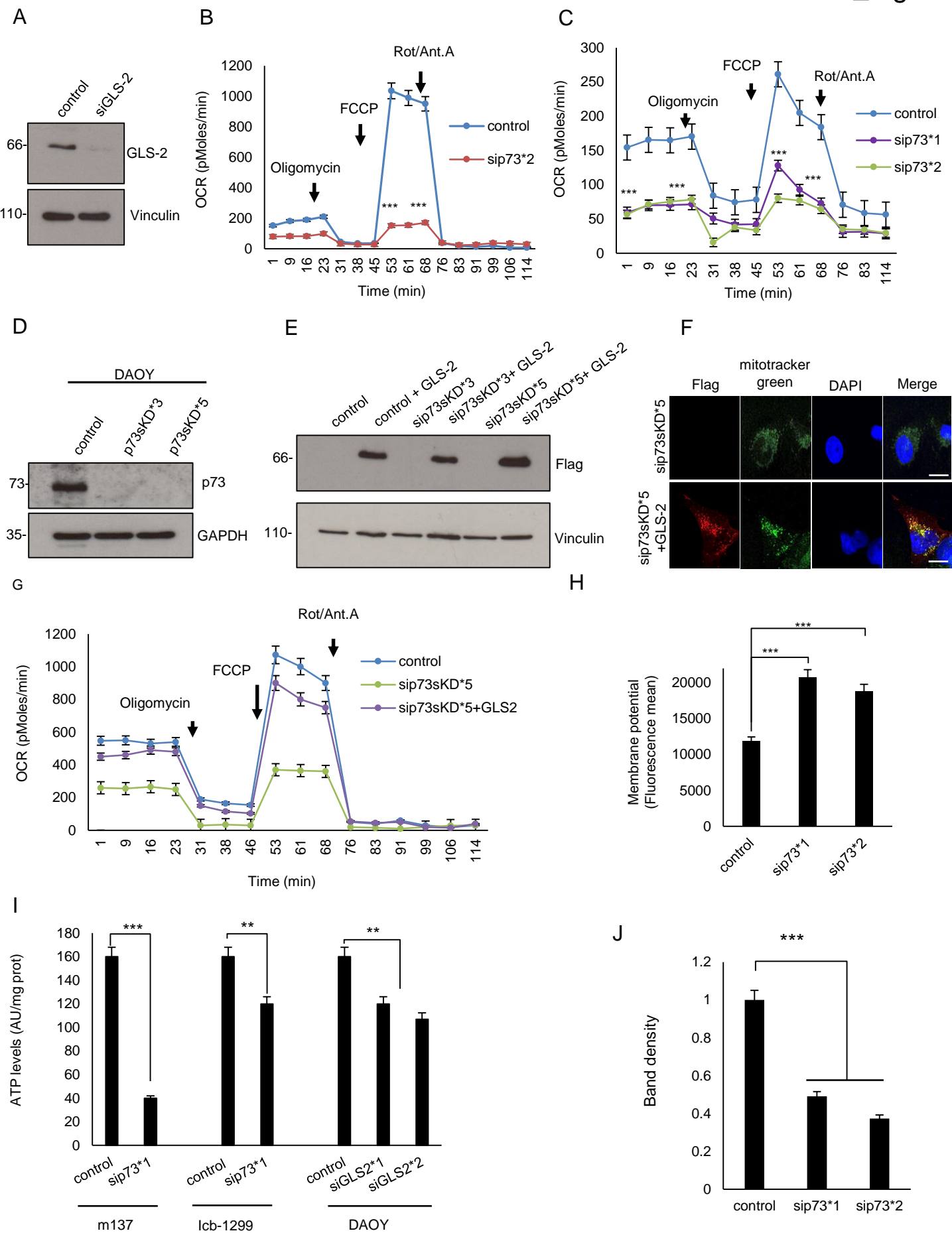

Supplement: Supplemental Material [file supp_gad.302349.117_Supplemental_Fig2.pdf]

A

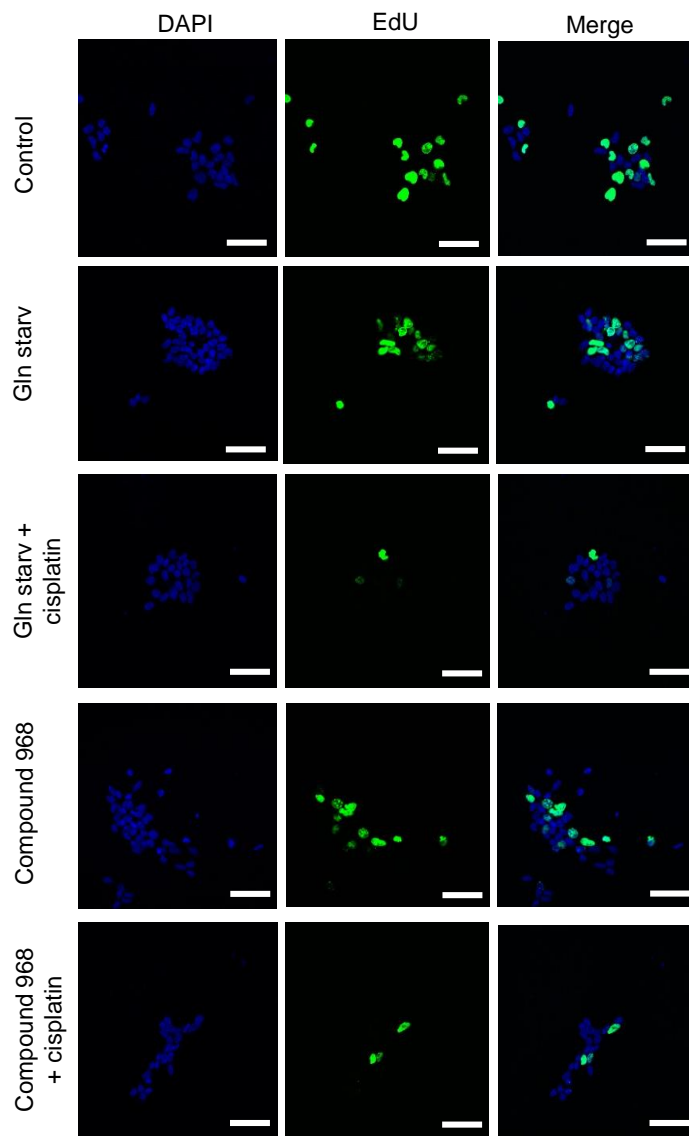

B

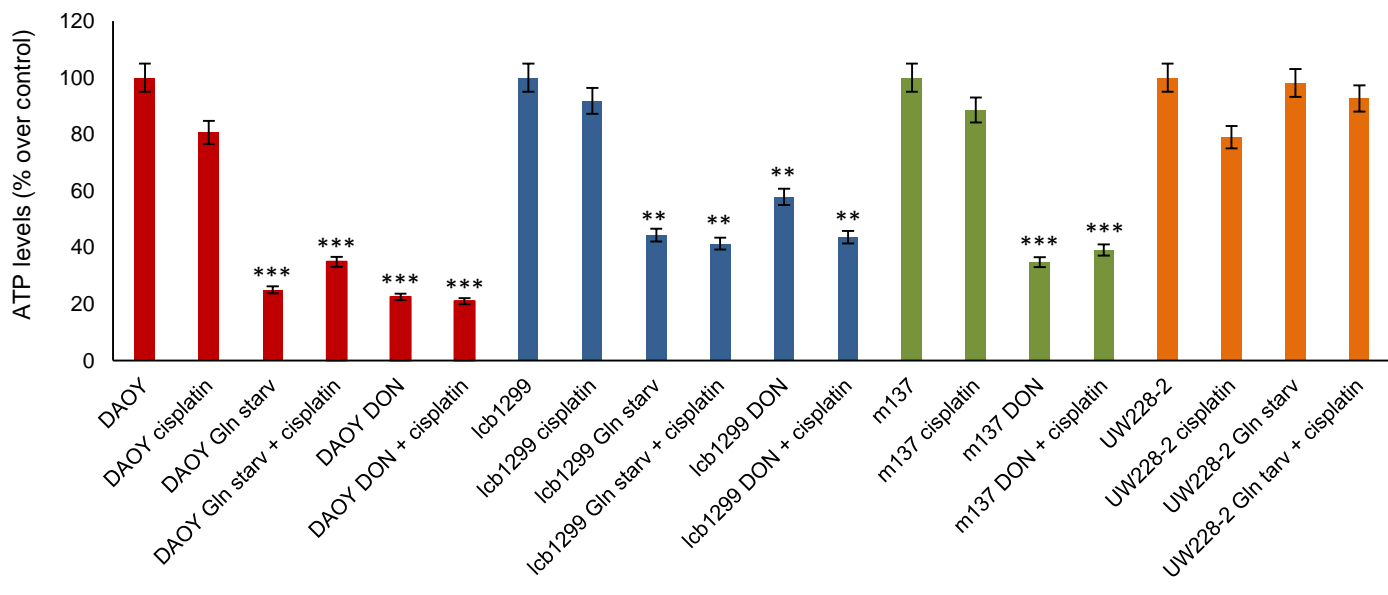

Supplement: Supplemental Material [file supp_gad.302349.117_Supplemental_Fig5.pdf]

A

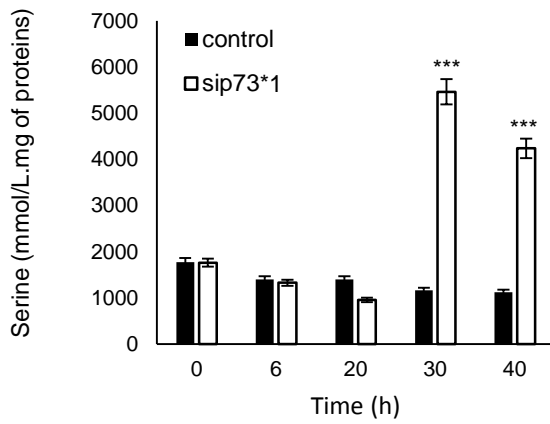

B

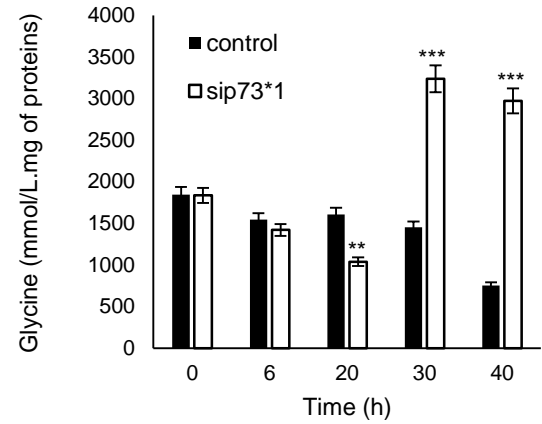

C

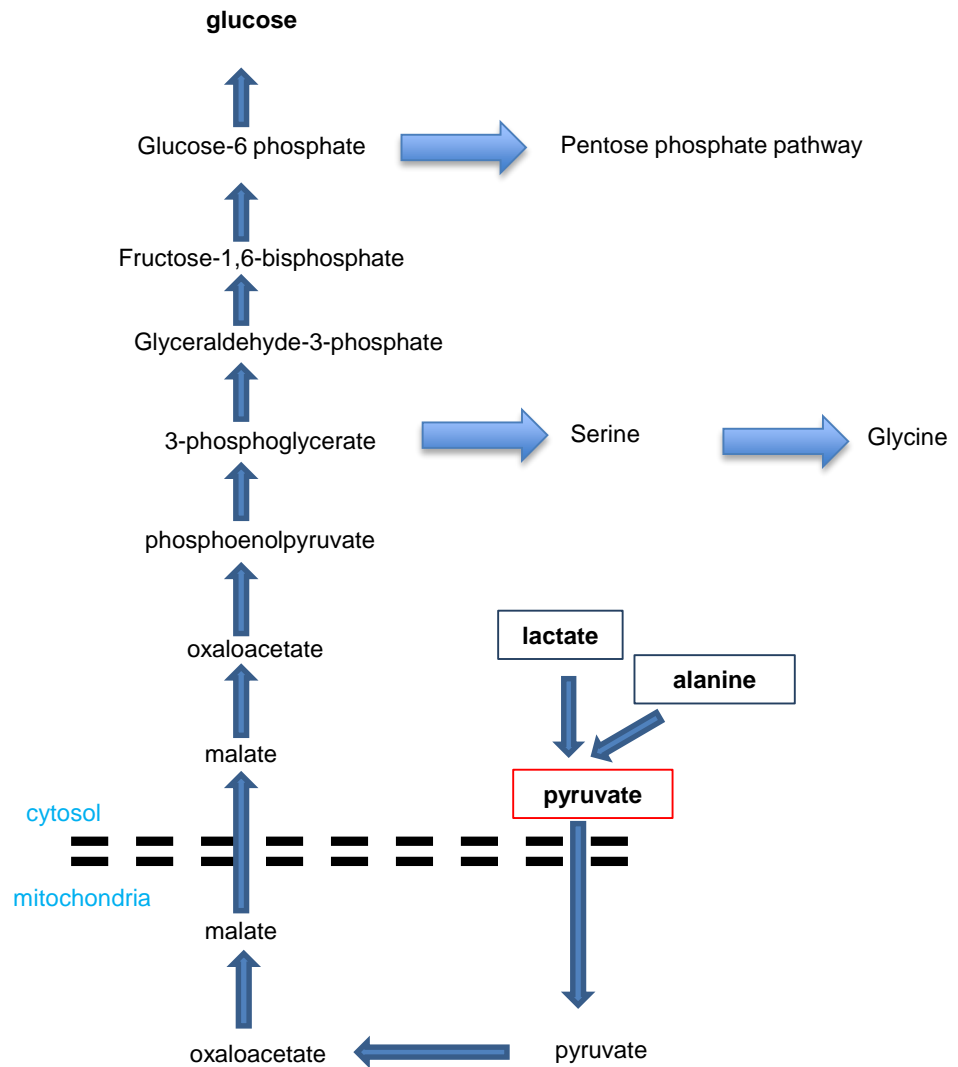

Supplement: Supplemental Material [file supp_gad.302349.117_Supplemental_Fig3.pdf]
