# Supplementary material for: TAp73 is a marker of glutamine addiction in medulloblastoma
Source: Genes Dev. 2017 Sep 1;31(17):1738–53. doi: 10.1101/gad.302349.117 (PMC5666673; doi:10.1101/gad.302349.117)
Supplement: Supplemental Material [file supp_gad.302349.117_Supplemental_Fig6.pdf]

A

	Geometric Mean		
	**12h**	**24h**	**12h + NAC**
DAOY Control	7.715 ± 0.4	15.26 ± 2.5	7.035± 0.5
DAOY Glc starv	32.966 ± 2.0	69.6 ± 27.8	14.85 ± 0.45
DAOY Gln starv	41.475 ± 12.2	158.33 ± 21.3	9.92 ± 2.58
DAOY Ser/Gly starv	47.325 ± 11.9	164.6 ± 27.5	5.175 ± 0.3
